# Fabrication of Dual Photodynamic Enhanced Antimicrobial CDs@ZIF‐8/Polycaprolactone/Ethyl Cellulose Nanofibrous Films for Fruit Preservation

**DOI:** 10.1002/advs.202503567

**Published:** 2025-06-23

**Authors:** Yongrui Ti, Ziqiang Qin, Xiaohong Guo, Zheng Wu, Qi Jiang, Beihua Dong, Olaniyi Amos Fawole, Di Wu, Su Chen, Xian Li, Kunsong Chen

**Affiliations:** ^1^ College of Agriculture & Biotechnology Zhejiang Key Laboratory of Horticultural Crop Quality Improvement Key Laboratory of Ministry of Agriculture and Rural Affairs of Biology and Genetic Improvement of Horticultural Crops (Growth and Development) Zhejiang University Hangzhou 310058 P. R. China; ^2^ College of Biosystems Engineering and Food Science Zhejiang University Hangzhou 310058 P. R. China; ^3^ Hualian Machinery Group Co., Ltd Wenzhou 325035 P. R. China; ^4^ Postharvest Research Laboratory Department of Botany and Plant Biotechnology University of Johannesburg P.O. Box 524, Auckland Park Johannesburg 2006 South Africa; ^5^ Zhejiang University Zhongyuan Institute Zhengzhou 450000 P. R. China; ^6^ State Key Laboratory of Materials‐Oriented Chemical Engineering & College of Chemical Engineering Jiangsu Key Laboratory of Fine Chemicals and Functional Polymer Materials Nanjing Tech University Nanjing 210009 P. R. China

**Keywords:** carbon dots, dual photodynamic enhanced antimicrobial, fruit, metal‐organic frameworks, microfluidic‐blow‐spinning, postharvest preservation

## Abstract

Carbon dots (CDs) have elicited attention in photodynamic antimicrobial applications, but their susceptibility to aggregation limits their effectiveness. Herein, CDs@ZIF‐8 nanocomposites are synthesized. The results reveal that they have superior dual photodynamic enhanced antimicrobial properties, specifically 7.63 log_10_ CFU mL^−1^ (lg) elimination for *Escherichia coli* (only 3.12 lg with CDs and 4.18 lg with ZIF‐8) and 7.27 lg elimination for *Staphylococcus aureus* (only 4.17 lg with CDs and 2.94 lg with ZIF‐8). Optoelectronic property analysis and density functional theory calculations show that the enhanced photodynamic antimicrobial properties of CDs@ZIF‐8 can be attributed to their narrow bandgap and higher binding affinity for O_2_, which facilitates the photo‐generated e^−^–h^+^ pairs separation and promotes the generation of ^1^O_2_ and •O_2_^−^. Furthermore, the CDs@ZIF‐8/polycaprolactone/ethyl cellulose nanofibrous films are prepared by microfluidic‐blow‐spinning, which have prominent antimicrobial properties and extend the shelf life of strawberries from 6 to 12 d. Overall, this study provides a novel idea for designing synergistically enhanced photodynamic antimicrobial materials and photodynamic preservation of perishable fruits.

## Graphical abstract

Carbon dots (CDs) are incorporated into ZIF‐8 to synthesize CDs@ZIF‐8 nanocomposites, which exhibit dual photodynamic enhanced antimicrobial properties. This enhancement is due to the narrow bandgap of CDs@ZIF‐8, which facilitates interfacial electronic transfer, resulting in increased reactive oxygen species. Microfluidic blow spinning is then used to prepare CDs@ZIF‐8/polycaprolactone/ethyl cellulose nanofibrous films, which exhibit good antimicrobial and fruit preservation properties.

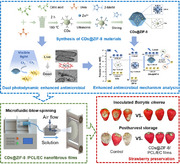

## 1 Introduction

Microbial contamination seriously threatens food, the environment, and public health, attracting widespread concern.^[^ ^1^ ^]^ Traditionally, chemical preservatives have been commonly used to control microbial growth, but their overuse leads to increased resistance, reduced effectiveness, and can compromise human health and environmental safety. According to the World Health Organization, microbial resistance will cause nearly 10 million deaths annually by 2050, making it a severe public health threat in the 21^st^ century.^[^ ^2^, ^3^ ^]^ Photodynamic antimicrobial has been used as an effective sterilizing technology to avoid the emergence of resistance, with green safety, efficient broad‐spectrum antimicrobial properties, low energy consumption, low cost, and no carcinogenic byproducts.^[^ ^4^, ^5^, ^6^, ^7^ ^]^ Therefore, photodynamic antimicrobial technology is expected to replace traditional chemical preservatives in controlling microbial contamination, with many promising applications. Nevertheless, studies on the structural design of photocatalytic materials and synergistic interactions between photosensitizers that promote reactive oxygen species (ROS) production for greater antimicrobial activity are scarce and there is still considerable room for optimization.^[^ ^8^, ^9^ ^]^

Carbon dots (CDs) are easy to prepare and have unique optical properties, low toxicity, good biocompatibility, and low costs. They are often referred to as the “ideal” carbon nanomaterial.^[^ ^10^, ^11^ ^]^ CDs have attracted great interest in photodynamic antimicrobial applications based on their unique advantages, such as small size (<10 nm), high quantum yield, broad excitation spectra, and efficient charge transfer rate.^[^ ^12^, ^13^ ^]^ In addition, CDs have wide applications in bioimaging, cell labeling, ion detection, anti‐counterfeiting, and optoelectronics.^[^ ^14^, ^15^ ^]^ Notably, the photodynamic antimicrobial activity of CDs is based on their unique photovoltaic properties and wide range of light‐trapping abilities, allowing them to act as photosensitizers to generate ROS under light to achieve antimicrobial activity.^[^ ^16^, ^17^ ^]^ However, CDs are prone to self‐aggregation, which limits their photosensitivity and antimicrobial effects, to some extent.^[^ ^18^ ^]^

Metal‐organic frameworks (MOFs) are excellent carriers for encapsulating various active molecules or nanoparticles.^[^ ^19^, ^20^ ^]^ Nevertheless, most MOFs show weak photocatalytic properties due to weak light absorption. Therefore, encapsulating CDs in MOFs to obtain hybrid nanostructures may be a feasible approach to improving the dispersion of CDs and the photocatalytic performance of CDs and MOFs. CDs can enhance the optoelectronic and light‐absorbing properties of MOFs. Furthermore, MOFs are porous and possess a high specific surface area. This provides an advantageous space for CDs encapsulation, which helps to avoid the self‐aggregation of CDs and increases their stability.^[^ ^21^ ^]^ ZIF‐8 has elicited great interest due to its ease of synthesis, large porosity, and tunable pore size.^[^ ^22^ ^]^ These advantages endow it with excellent application prospects for loading CDs to exert enhanced photodynamic antimicrobial properties. Given this, combining CDs with ZIF‐8 is expected to create highly efficient dual photodynamic enhanced antimicrobial materials. However, previous studies on combing of CDs and MOFs have mainly focused on fluorescence sensing,^[^ ^23^ ^]^ biological analysis,^[^ ^24^ ^]^ and LED devices,^[^ ^25^ ^]^ and no research has been reported on the synergistic enhancement of the photodynamic antimicrobial properties of both CDs and ZIF‐8.

Fresh fruits suffer from severe postharvest losses, with the main cause being microbial infections.^[^ ^26^, ^27^ ^]^ Packaging is an essential means of reducing postharvest losses and preserving fruit quality, and it performs a vital function during storage, transportation, and sale. Nanofibers possess specific high surface areas, large porosity, excellent mechanical properties, and adjustable structures. Given these advantages, nanofibrous films have good potential for application in fruit preservation packaging. Microfluidic‐blow‐spinning (MBS) technology can produce nanofibers with high porosity and high surface area. Compared with electrospinning, MBS technology offers advantages including faster processing, energy efficiency, and safety, and has been applied in fruit and vegetable preservation research.^[^ ^28^, ^29^, ^30^ ^]^ However, there are no studies on the preparation of dual photodynamic enhanced antimicrobial nanofiber packaging using MBS technology.

In the present study, a novel dual photodynamic enhanced antimicrobial nanofiber packaging material was developed to address the substantial postharvest loss of perishable fruits caused by microbial infections. First, CDs@ZIF‐8 composites were synthesized by incorporating CDs into ZIF‐8 cavities through self‐assembly. The structure, photodynamic antimicrobial properties, and mechanism of the synthesized materials were then evaluated. Subsequently, the CDs@ZIF‐8 nanocomposites were spun with polycaprolactone (PCL) and ethyl cellulose (EC) using MBS technology to prepare nanofibrous films with light‐responsive antimicrobial properties (CDs@ZIF‐8/PCL/EC). The physicochemical properties and photodynamic antimicrobial activity of the nanofibrous films were also evaluated. Lastly, using strawberries as a representative example, the potential application of nanofibrous films in preserving perishable fruits was investigated. This research aims to develop synergistic photodynamic enhanced antimicrobial materials for the preservation of perishable foods to reduce waste.

## 2 Results and Discussion

### 2.1 Characterization of CDs@ZIF‐8

**Figure 1:**
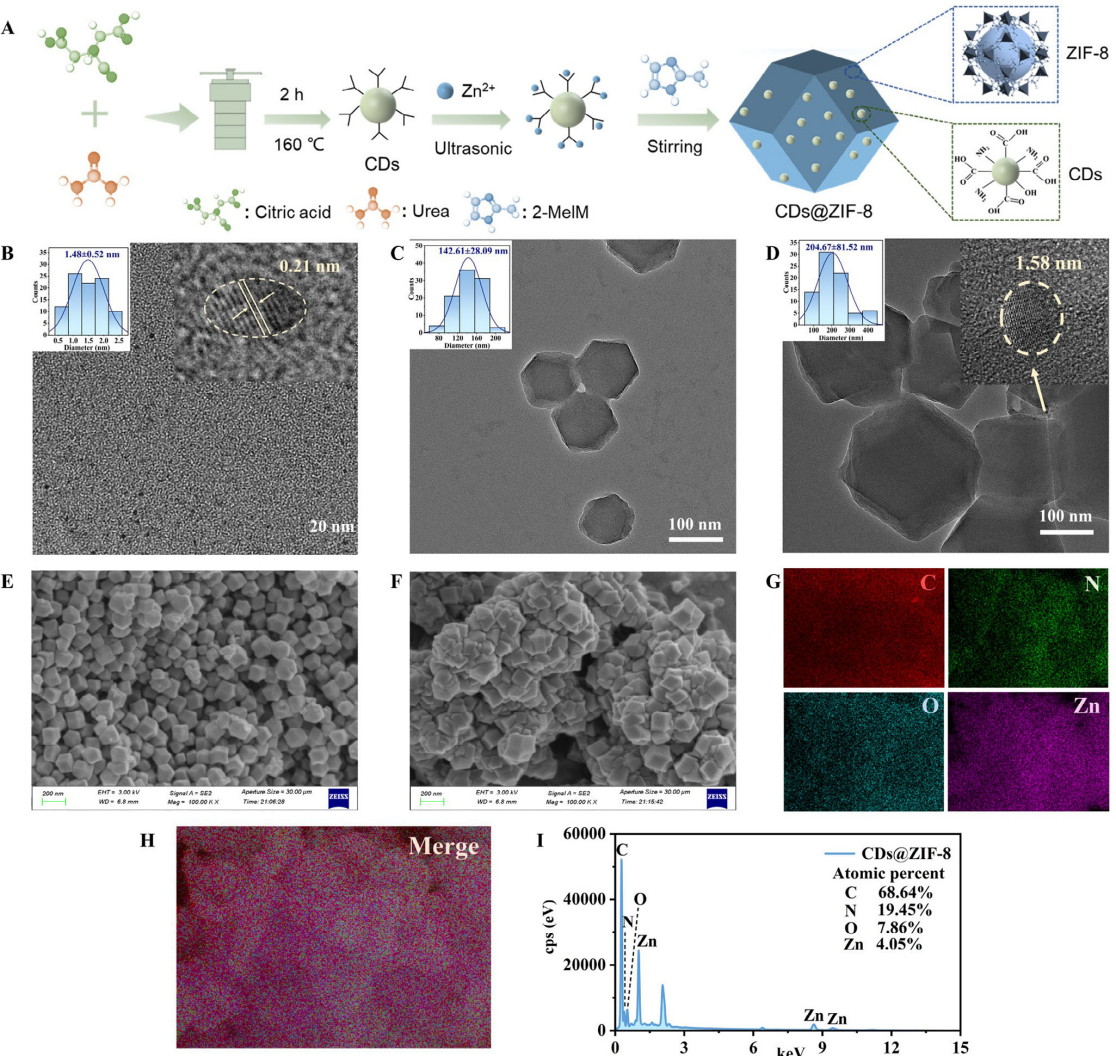
A) CDs@ZIF‐8 nanocomposites preparation process. TEM micrographs of B) CDs, C) ZIF‐8, and D) CDs@ZIF‐8 nanocomposites. SEM micrographs of E) ZIF‐8 and F) CDs@ZIF‐8 nanocomposites. G–I) EDX spectral mapping of CDs@ZIF‐8 nanocomposites.

CDs were synthesized using the hydrothermal method. Subsequently, the pre‐synthesized CDs were dispersed into a mixed solution consisting of Zn^2+^ and 2‐methylimidazole (2‐MeIM) precursor, and the CDs@ZIF‐8 nanocomposites were prepared through coordination self‐assembly (**Figure** **1**A). The synthesized CDs exhibited a spherical morphology with an average particle size of 1.48±0.52 nm (Figure 1B). Notably, the lattice spacing of the CDs was 0.21 nm, corresponding to the d‐spacing (100) crystal plane of graphene, indicating that the synthesized CDs had a graphene‐like structure.^[^ ^31^ ^]^ ZIF‐8 displayed a dodecahedral structure (Figure 1C), which was consistent with the SEM image (Figure 1E and Figure S1, Supporting Information). The average particle size of ZIF‐8 was 142.61±28.09 nm from the particle size dispersion plot. The morphology of the CDs@ZIF‐8 nanocomposites was essentially identical to that of ZIF‐8 (Figure 1D,F). Encapsulating the CDs increased their particle sizes, with CDs@ZIF‐8 exhibiting an average size of 204.67±81.52 nm (Figure 1D). These findings agree with the results of Wang et al.,^[^ ^32^ ^]^ who reported that incorporating of quantum dots increased the particle size of ZIF‐8. The elemental distributions of C, N, and O in the CDs were relatively uneven, which may be due to the aggregation tendency of CDs (Figure S2, Supporting Information).^[^ ^25^ ^]^ ZIF‐8 contained C, N, and Zn elements with a uniform distribution (Figure S3, Supporting Information). In the CDs@ZIF‐8 nanocomposites, C, N, O, and Zn elements were uniformly distributed (Figure 1G–I), indicating that the CDs were almost not aggregated within the ZIF‐8 framework, thereby improving their dispersion.

**Figure 2:**
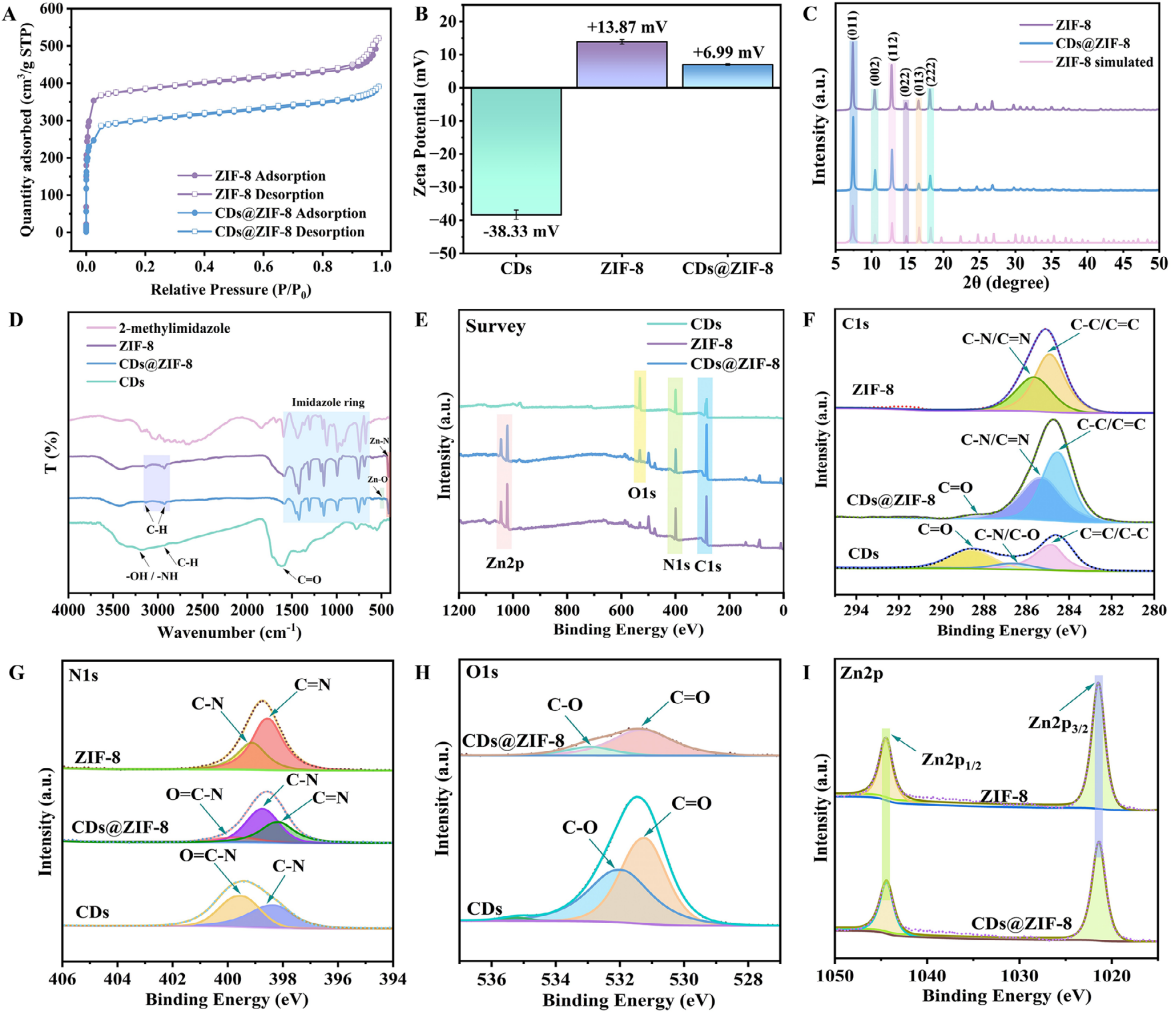
A) N_2_ sorption isotherm curves. B) Zeta potential. C) XRD spectra. D) FT‐IR spectra. E) XPS survey spectra, F) C1s, G) N1s, H) O1s, and I) Zn2p high‐resolution spectra.

The CD, ZIF‐8 and CDs@ZIF‐8 solutions were dark brown, white, and brown, respectively (Figure S4, Supporting Information). The CDs emitted blue‐green fluorescence under irradiation at a wavelength of 365 nm, confirming their successful synthesis (Figure S5, Supporting Information). Fluorescence spectra showed that the maximum emission wavelengths for the CDs and CDs@ZIF‐8 were approximately 443 nm and 586 nm, having undergone a significant redshift (Figure S6, Supporting Information). The results may be related to a close interfacial interaction between ZIF‐8 and CDs. As shown in **Figure** **2**A, the N_2_ adsorption‐desorption isotherms of pristine ZIF‐8 and CDs@ZIF‐8 exhibited typical type I isotherms. The Brunauer‐Emmett‐Teller (BET) specific surface area of pristine ZIF‐8 was 1576.713 m^2^ g^−1^, whereas the N_2_ adsorption capacity of CDs@ZIF‐8 was slightly less than that of pristine ZIF‐8, and the BET specific surface area was 1202.078 m^2^ g^−1^. This was mainly attributed to CDs occupying the internal space of ZIF‐8. Nevertheless, the BET specific surface area remained relatively high. Moreover, the pore width distributions of both ZIF‐8 and CDs@ZIF‐8 ranged from 0.4 to 1.4 nm, indicating that incorporating CDs did not significantly affect the microporous structure of ZIF‐8 (Figure S7, Supporting Information). The zeta potential values of the CDs, ZIF‐8, and CDs@ZIF‐8 was ‐38.33 mV, +13.87 mV, and +6.99 mV, respectively (Figure 2B). The zeta potential of CDs@ZIF‐8 was closer to that of ZIF‐8 nanoparticles than CDs, demonstrating that most CDs were successfully incorporated into ZIF‐8.

The XRD spectrum of ZIF‐8 demonstrated crystalline characteristic signals corresponding to the crystalline planes of simulated ZIF‐8, confirming its successful synthesis (Figure 2C).^[^ ^33^ ^]^ Remarkably, the XRD spectrum of CDs@ZIF‐8 was almost identical to that of pristine ZIF‐8, demonstrating that the encapsulated CDs did not affect the crystal structure of ZIF‐8. Figure 2D shows the FT‐IR of the prepared sample, the peak at 3350 cm^−1^ in the CDs was associated with the stretching vibrations of ─OH/─NH. The peak at 2930 cm^−1^ corresponded to the stretching vibrations of ─CH. The peak at 1627 cm^−1^ was associated with C═O stretching vibrations.^[^ ^24^, ^34^ ^]^ The results show that the CDs were enriched with carboxyl and amino groups, facilitating metal ion binding and ensuring good water solubility. Typical peaks in a range of 600–1500 cm^−1^ were assigned to imidazole ring vibrations, and the peaks at 3140 cm^−1^ and 2930 cm^−1^ were due to ─CH_2_/CH_3_ stretching vibrations.^[^ ^35^ ^]^ Notably, a new peak at 496 cm^−1^ in the CDs@ZIF‐8 nanocomposites resulted from Zn─O bond stretching vibrations.^[^ ^36^, ^37^ ^]^ Furthermore, the peak at 420 cm^−1^ was associated with Zn─N bond stretching vibrations.^[^ ^38^ ^]^ The results indicate that the ─COOH and ─NH_2_ groups of CDs could coordinate with Zn^2+^ to form Zn─O and Zn─N bonds, which was critical for embedding the CDs in the ZIF‐8 cavities.^[^ ^39^ ^]^ In addition, the peak intensity of the characteristic functional groups of the CDs was attenuated in CDs@ZIF‐8, indicating the efficient encapsulation of most CDs in ZIF‐8 cavities.

The elemental details of the prepared samples were investigated using XPS spectra. Figure 2E shows Zn, C, O, and N in the CDs@ZIF‐8 nanocomposites. As indicated by the C1s spectrum (Figure 2F), C1s at 284.8 eV, 286.4 eV, and 288.3 eV in CDs were due to C─C/C═C, C─O/C─N, and C═O bonds, respectively.^[^ ^24^, ^34^ ^]^ The peaks at 284.8 eV and 285.6 eV were attributed to the C─C/C═C and C─N/C═N bonds of 2‐MeIM in ZIF‐8. Three peaks at approximately 284.8 eV, 285.6 eV, and 288.3 eV were observed in the CDs@ZIF‐8 nanocomposites. The extra peak, compared with ZIF‐8, at 288.3 eV contributed to the C═O bond in the CDs.^[^ ^40^ ^]^ Notably, the combination of CDs with ZIF‐8 greatly decreased the C═O configuration in the CDs@ZIF‐8 nanocomposites compared with that of the CDs, demonstrating that CDs were effectively embedded in ZIF‐8. High‐resolution N1s is shown in Figure 2G, the XPS fitting peaks of N1s from CDs were roughly separated into two characteristic peaks at 398.6 and 400.3 eV, probably originating from C─N bonds and ─O═C─NH─ bonds.^[^ ^40^ ^]^ ZIF‐8 exhibited two peaks at 398.62 eV and 399.2 eV, corresponding to C═N and C─N bonds.^[^ ^35^ ^]^ The extra peak, compared with ZIF‐8, at 400.3 eV in the CDs@ZIF‐8 nanocomposites was due to ─O═C─NH─ bonds in the CDs. In the high‐resolution spectrum of the O1s (Figure 2H), two prominent peaks were observed in the CDs and CDs@ZIF‐8, which were assigned to C═O and C─O bonds.^[^ ^31^, ^41^ ^]^ Figure 2I shows that the Zn2p fitted peaks for ZIF‐8 and CDs@ZIF‐8 displayed similar characteristics. Interestingly, in the C1s, N1s and O1s spectra of the CDs@ZIF‐8 nanocomposites, the locations of the C─N and C─O peaks showed slight shifts in contrast to the ZIF‐8 and CDs. The slight change in peak location may be associated with the formation of interfacial bonds (Zn─O and Zn─N),^[^ ^20^ ^]^ which was consistent with the FT‐IR and fluorescence spectroscopy results.

### 2.2 Antimicrobial Activity of CDs@ZIF‐8

**Figure 3:**
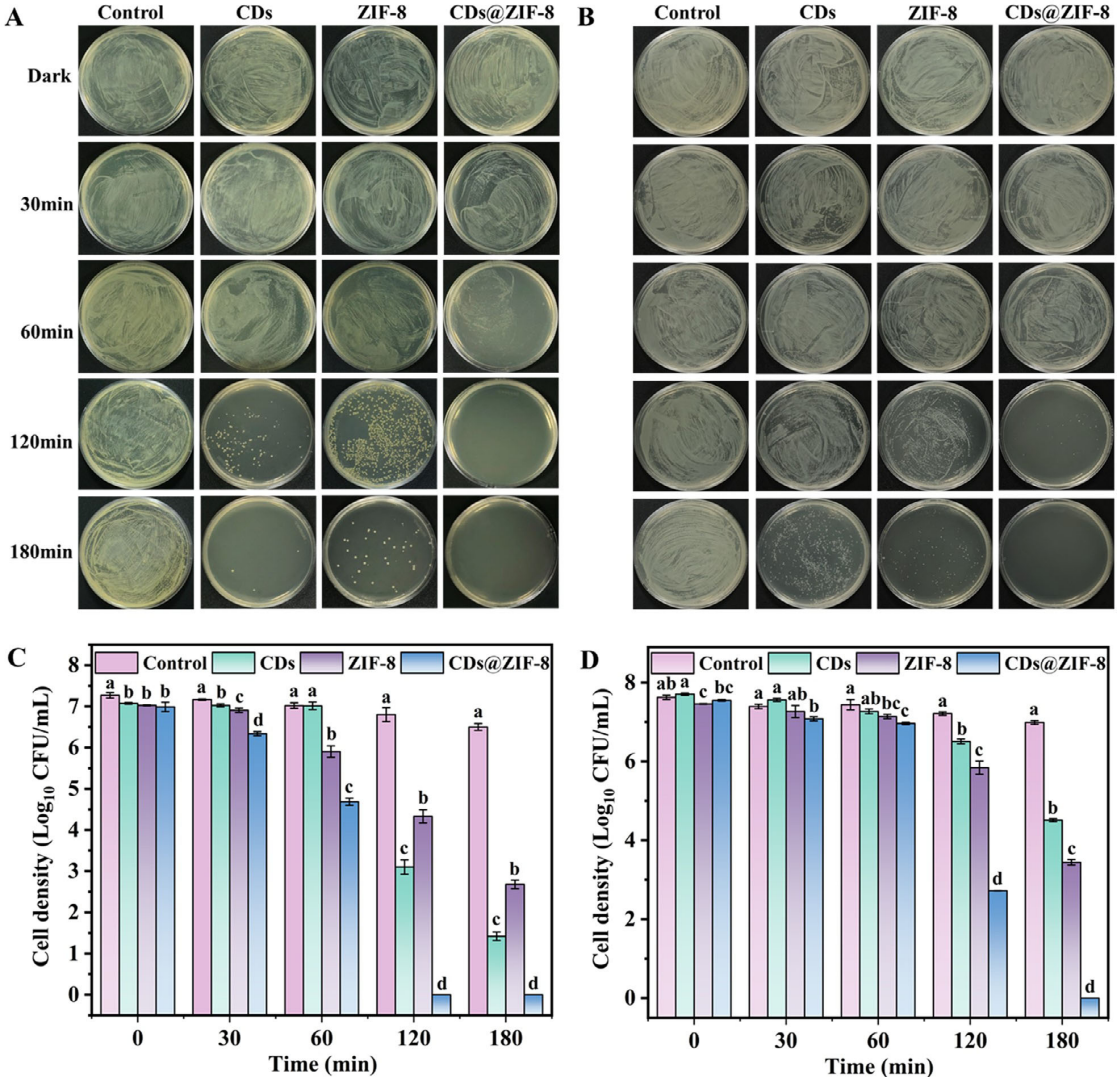
A) *S. aureus* and B) *E. coli* antimicrobial experiment at different light exposure times. Plots of changes in C) *S. aureus* and D) *E. coli* counts (*n* = 3 in each group). Different letters (a–g) represent significant differences (*p < 0.05).

Microbial growth accelerates fruit spoilage, reducing their edible and commercial value and causing significant economic losses. This study investigated the photodynamic antimicrobial activity of synthesized nanomaterials against *Staphylococcus aureus* (*S. aureus*), *Escherichia coli* (*E. coli*), and *Botrytis cinerea* (*B. cinerea*). **Figure** **3**A shows that the CDs, ZIF‐8, and CDs@ZIF‐8 nanocomposites exhibited weak antimicrobial activity under dark conditions. Nevertheless, the CDs@ZIF‐8 nanocomposites completely inhibited the growth of *S. aureus*, achieving a 7.27 log_10_ CFU mL^−1^ (lg) reduction when exposed to visible light for 120 min (Figure 3C). This value was markedly higher than that of CDs (4.17 lg reduction) and ZIF‐8 (2.94 lg reduction) alone (Table S1, Supporting Information). Similarly, the CDs, ZIF‐8, and CDs@ZIF‐8 nanocomposites had weak antimicrobial activity against *E. coli* in the dark (Figure 3B). However, the CDs@ZIF‐8 nanocomposites achieved a 7.63 lg reduction in *E. coli* colonies, which was significantly higher than that of CDs (3.12 lg reduction) and ZIF‐8 (4.18 lg reduction) after exposure to visible light for 180 min (Figure 3D and Table S1, Supporting Information). Moreover, the CDs@ZIF‐8 nanocomposites effectively eliminated 5 lg of *B. cinerea* spores and exhibited dose‐dependent photodynamic antimicrobial activity (Figures S8 and S9, Supporting Information). The higher antimicrobial concentration of CDs@ZIF‐8 against *B. cinerea* compared to *S. aureus* and *E. coli* might be attributed to the different cell structures of fungi and bacteria.^[^ ^42^ ^]^ In addition, the photodynamic antimicrobial activity of CDs@ZIF‐8 nanocomposites was better than that of the mechanically mixed CDs/ZIF‐8 nanocomposites (Figure S10, Supporting Information). These results suggest that the CDs@ZIF‐8 nanocomposites exhibit enhanced dual photodynamic antimicrobial activity, making them a promising candidate for applications in antimicrobial treatment and food preservation.

Photostability is a key factor in ensuring the long‐term effectiveness of photodynamic antimicrobial materials. The photodynamic antimicrobial time was 2 h for *S. aureus* and 3 h for *E. coli*. Based on this, 5–30 h of continuous light was chosen to evaluate the stability of CDs@ZIF‐8 under light conditions. Specifically, the CDs@ZIF‐8 nanocomposites were first exposed to different light durations (5–30 h). Then, their antimicrobial activity was measured by co‐culture with the corresponding bacteria (3 h for *E. coli* and 2 h for *S. aureus*) under visible light. The results show that the CDs@ZIF‐8 nanocomposites still had good antimicrobial performance after different light durations (5–30 h) (Figure S11A, Supporting Information). Even after 30 h of continuous irradiation, 7.80 lg of *E. coli* was effectively eliminated after 3 h of photodynamic treatment and 7.58 lg of *S. aureus* were effectively eliminated after 2 h of photodynamic treatment (Figure S11B, Supporting Information), resulting in no bacterial colonies growing on the solid medium. The results indicate that the CDs@ZIF‐8 nanocomposites had good stability under light conditions.

### 2.3 Reactive Oxygen Species Determination

**Figure 4:**
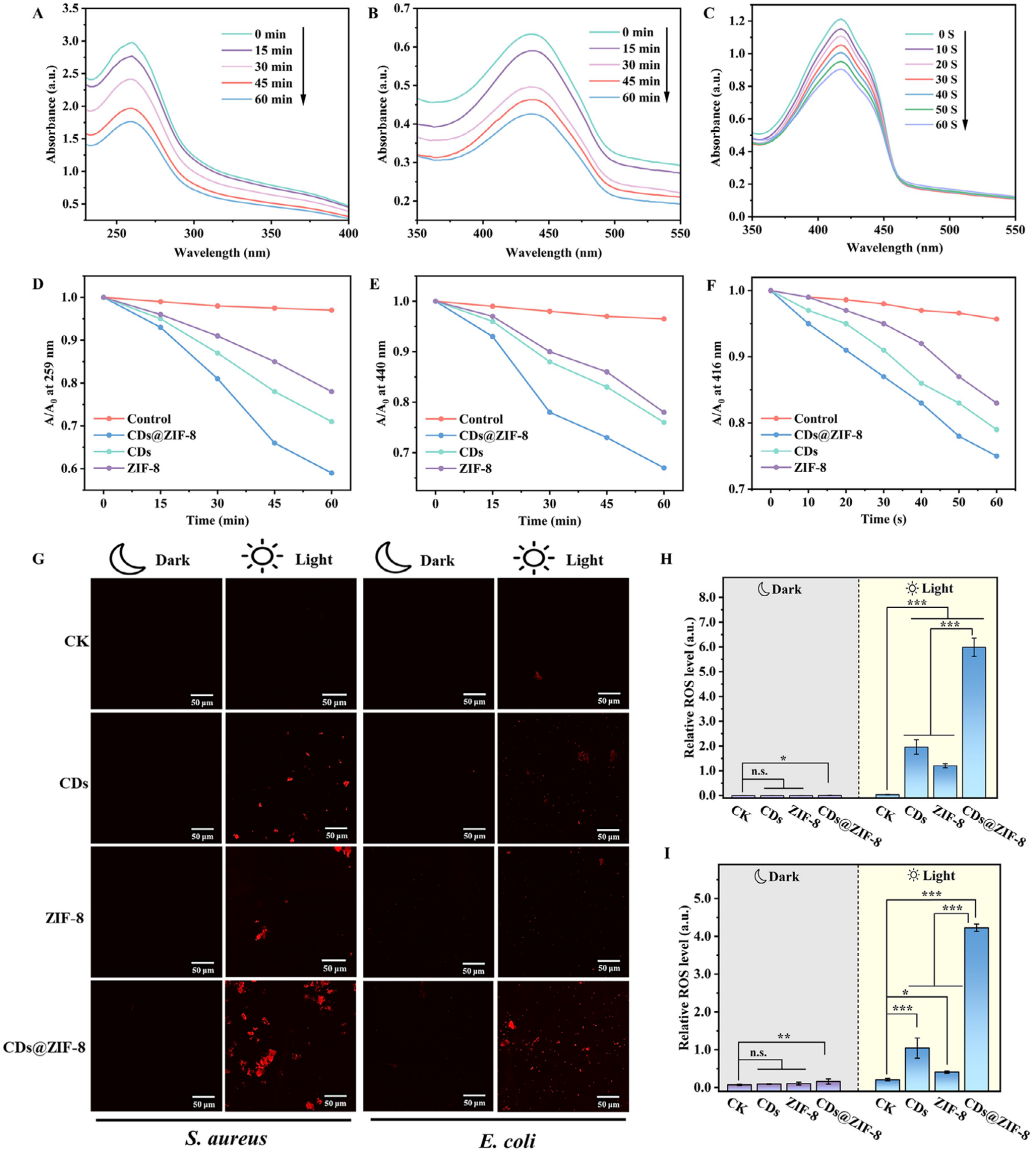
A) Determination of •O_2_^−^ using NBT absorption, B) ^1^O_2_ using RNO absorption and C) ^1^O_2_ using a DPBF fluorescent probe in the CDs@ZIF‐8 treatment under light. The photooxidation rates of D) NBT, E) RNO, and F) DPBF in different treatment groups. G) ROS in *S. aureus* and *E. coli* under light and dark (scale bar = 50 µm). Quantification of ROS levels in H) *S. aureus* and I) *E. coli* cells (*n* = 3). *p < 0.05, **p < 0.01, and ***p < 0.001; n.s. indicates no significant difference.

CDs@ZIF‐8 nanocomposites exhibited enhanced dual‐photodynamic antimicrobial properties, due to their ability to produce large amounts of ROS under light. The superoxide anion radical (•O_2_^−^) was monitored based on its reaction with nitroblue tetrazolium (NBT) to form formazan, which reduces the absorbance peak of NBT at 259 nm. The decrease in this absorbance peak correlates with •O_2_^−^ production.^[^ ^43^ ^]^ **Figure** **4**A shows a gradual decrease in NBT absorbance with prolonged light exposure, indicating that the CDs@ZIF‐8 nanocomposites generated •O_2_^−^ under light. Notably, the rate of •O_2_^−^ generation was significantly higher in the CDs@ZIF‐8 than in the CDs or ZIF‐8 alone (Figure 4D). In contrast, the change in NBT absorbance under dark conditions was almost negligible, indicating that •O_2_^−^ was essentially not produced under dark conditions (Figure S12A, Supporting Information). Similarly, the production of singlet oxygen (^1^O_2_) was determined using the p‐nitrosodimethylaniline (RNO) absorption spectrum. The peroxide intermediate RNO formed during the reaction of imidazole and ^1^O_2_ had a bleaching effect, resulting in a decrease in absorbance at 440 nm.^[^ ^44^ ^]^ The absorption at 440 nm gradually declined with the illumination time, demonstrating that the CDs@ZIF‐8 nanocomposites could generate ^1^O_2_, and that the rate of ^1^O_2_ generation was significantly higher than that of CDs or ZIF‐8 alone (Figure 4B,E). Under dark conditions, the absorbance of RNO remained essentially unchanged with time, suggesting that the CDs@ZIF‐8 nanocomposites did not produce ^1^O_2_ in the dark (Figure S12B, Supporting Information). Further ROS detection was performed using the fluorescence probe 1,3‐diphenylisobenzofuran (DPBF), which is highly specific to ^1^O_2_. Upon binding with^1^O_2_, DPBF underwent irreversible oxidation, decreasing in absorbance at 416 nm.^[^ ^45^ ^]^ Figure 4C,F shows that the absorbance intensity of DPBF decreased rapidly with prolonged light exposure, indicating ^1^O_2_ production by CDs@ZIF‐8 nanocomposites. The photooxidation rate of DPBF in CDs@ZIF‐8 was significantly faster than that of the CDs and ZIF‐8. By contrast, there was essentially no ^1^O_2_ generation under dark conditions (Figure S12C, Supporting Information). These results reveal that the CDs@ZIF‐8 nanocomposites could produce more •O_2_^−^ and ^1^O_2_ and that the ROS production rates were markedly higher than those using CDs and ZIF‐8 alone under light.

Intracellular ROS levels were then assessed in bacterial cells treated with CDs@ZIF‐8 nanocomposites. Dihydroethidium (DHE) diffuses easily across the cell membrane and is oxidized by intracellular ROS to generate oxyethidium. Oxyethidium can integrate into chromosomal DNA, resulting in red fluorescence. The intensity of this fluorescence directly correlates with the ROS levels in bacterial cells. Figure 4G shows that under light conditions, the CDs, ZIF‐8, and CDs@ZIF‐8 groups exhibited red fluorescence in *S. aureus* and *E. coli* cells, indicating their ability to induce ROS production in bacterial cells and demonstrating photodynamic antimicrobial activity. In contrast, under dark conditions, the red fluorescence in *S. aureus* and *E. coli* cells was relatively weak and nearly undetectable, indicating that ROS production was almost nonexistent in the dark. This observation was consistent with the in vitro ROS determination results. As shown in Figure 4H,I, the fluorescence was relatively weak for CDs and ZIF‐8 under light conditions. Nevertheless, the CDs@ZIF‐8 nanocomposites group exhibited significantly enhanced red fluorescence under light (*p* < 0.001). The results show that binding CDs and ZIF‐8 could induce higher ROS levels, thereby enhancing their photodynamic antimicrobial performance.

### 2.4 Bacterial Morphology Analysis

ROS, are known for their strong oxidative capacity, are reported to damage the membranes of bacteria, ultimately causing lysis and death.^[^ ^46^ ^]^ Therefore, the change in bacterial morphology was assessed to reveal the photodynamic antimicrobial performance of the CDs@ZIF‐8 nanocomposites. In the control and dark treatment groups, *S. aureus* and *E. coli* retained typical spherical and rod structures, respectively (Figure S13, Supporting Information). The bacterial cell membranes were relatively intact, and no cell membrane rupture was observed in the image. In contrast, after treatment with CDs, ZIF‐8 and the CDs@ZIF‐8 nanocomposites in the light, the bacterial cell membranes were somewhat damaged, resulting in cellular content leakage. Notably, the CDs@ZIF‐8 nanocomposites exhibited more pronounced membrane rupture and leakage of cellular contents treated in both *S. aureus* and *E. coli* than those of CDs and ZIF‐8. This suggests that the CDs@ZIF‐8 nanocomposites may be more effective in disrupting bacterial integrity, ultimately leading to bacterial lysis and death.^[^ ^47^ ^]^ These results were consistent with the plate count results, confirming that the CDs@ZIF‐8 nanocomposites exhibited better dual enhanced photodynamic antimicrobial activity than CDs and ZIF‐8 alone.

### 2.5 Mechanism for Dual Photodynamic Enhanced Antimicrobial Activity

**Figure 5:**
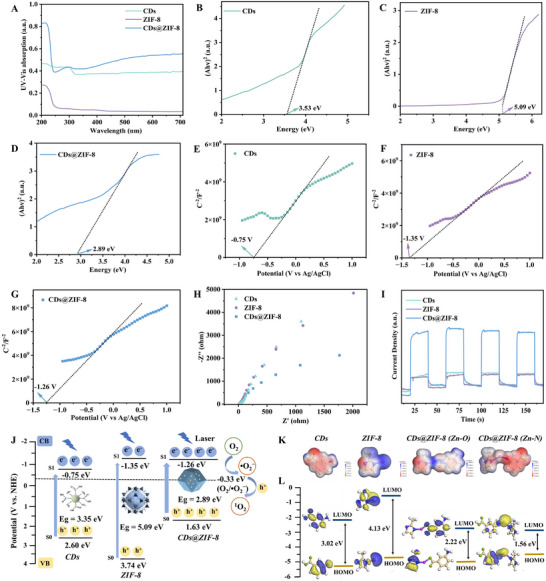
A) UV–vis DRS spectra. Bandgaps plots of B) CDs, C) ZIF‐8, D) CDs@ZIF‐8. Mott‐Schottky plots of E) CDs, F) ZIF‐8, and G) CDs@ZIF‐8. H) EIS Nyquist plots. I) Transient photocurrent response spectra. J) Energy level diagram of CDs, ZIF‐8, and CDs@ZIF‐8. K) Charge distribution and L) HOMO and LUMO orbital distribution from DFT calculations.

To investigate the mechanism of the dual photodynamic enhanced antimicrobial activity caused by CDs and ZIF‐8, the light absorption properties, photoluminescence spectra, ultrafast transient absorption (TA) spectroscopy, and the energy band structure were determined. As shown in **Figure** **5**A, the visible light absorption of ZIF‐8 was low. Notably, the absorption of CDs@ZIF‐8 was significantly enhanced compared with ZIF‐8 and CDs, leading to the more efficient use of visible light in the photocatalytic process, resulting in more photo‐generated carriers involved in the photocatalytic reaction. The photoluminescence signal of CDs@ZIF‐8 was diminished compared with ZIF‐8 and CDs (Figure S6, Supporting Information), suggesting that the combined CDs and ZIF‐8 effectively inhibit the recombination of photo‐generated e^−^–h^+^ pairs. Besides, the TA spectra of the CDs, ZIF‐8, and CDs@ZIF‐8 nanocomposites were recorded across probe delay times ranging from 1 to 3000 ps (Figure S14A–C, Supporting Information). A negative ΔA signal corresponding to ground‐state bleaching (GSB) was observed for all samples.^[^ ^48^ ^]^ The time profiles of the TA kinetics at 450 nm were fitted using a biexponential decay model. Notably, the CDs@ZIF‐8 nanocomposites demonstrated longer lifetimes than those of ZIF‐8 or CDs alone (Figure S14D–F, Supporting Information). The prolonged lifetimes in CDs@ZIF‐8 nanocomposites directly confirmed that they could more effectively inhibit the recombination of photogenerated carriers, facilitating the accumulation of photogenerated electrons.^[^ ^49^, ^50^ ^]^ Furthermore, the bandgaps (Eg) of the CDs, ZIF‐8 and CDs@ZIF‐8 were 3.53, 5.09, and 2.89 eV, respectively (Figure 5B–D). The bandgap of CDs@ZIF‐8 was lower than ZIF‐8 and CDs, and the reduced bandgap led to more efficient photo‐generated e^−^–h^+^ pairs separation.^[^ ^51^ ^]^ Moreover, Mott‐Schottky analysis shows that the CDs, ZIF‐8 and CDs@ZIF‐8 were all n‐type semiconductors,^[^ ^52^ ^]^ with flat band potentials of ‐0.75, ‐1.35 and ‐1.26 eV (vs Ag/AgCl), respectively (Figure 5E–G). Studies have reported that the conduction band potential of n‐type semiconductors was typically 0.1–0.2 V higher than the flat band potential.^[^ ^53^ ^]^ Herein, we assumed the potential difference between the flat and conduction band value to be 0.2 V. According to *E*(NHE) = *E*(Ag/AgCl)+0.197, the conduction band potentials of the CDs, ZIF‐8 and CDs@ZIF‐8 with respect to NHE were ‐0.75, ‐1.35 and ‐1.26 eV, respectively.^[^ ^54^ ^]^ Furthermore, based on *E*_VB_ = *E*_CB_ + *E*_g_, the valence band values of CDs, ZIF‐8 and CDs@ZIF‐8 were 2.60, 3.74 and 1.63 eV, respectively (compared with NHE; Figure 5J). Electrochemical impedance spectroscopy (EIS) measurements are shown in Figure 5H, where the arc radius of the CDs@ZIF‐8 nanocomposites was significantly lower than those of ZIF‐8 and CDs, indicating that the CDs@ZIF‐8 nanocomposites had lower interfacial charge transfer resistances, allowing for higher charge transfer efficiency during photodynamic antimicrobial processes. In addition, the CDs@ZIF‐8 nanocomposites exhibited a higher transient photocurrent response than ZIF‐8 and CDs (Figure 5I), further indicating excellent photo‐generated carriers separation efficiency.^[^ ^55^ ^]^

Density functional theory (DFT) calculations were then performed to elucidate the synergistically enhanced antimicrobial mechanism of CDs and ZIF‐8, focusing on charge distribution, electron transfer, highest occupied molecular orbital (HOMO), lowest occupied molecular orbital (LUMO), and binding energy to the O_2_ of CDs, ZIF‐8, CDs@ZIF‐8. Figure 5K shows that the positive charges in ZIF‐8 were mainly concentrated near the Zn atom. In the CDs, the negative charge was mainly concentrated on the O and N atoms, indicating that CDs and ZIF‐8 were more likely to form Zn─O and Zn─N bonds during coordination self‐assembly. This corresponded to the structural characterization and electron transfer calculation results (Table S2, Supporting Information). Figure 5L shows that in CDs@ZIF‐8, the LUMO was mainly located at the Zn─O and Zn─N binding sites of ZIF‐8 and CDs, forming a donor–acceptor system.^[^ ^56^ ^]^ Furthermore, the calculation of HOMO and LUMO levels in Figure 5L and Table S3 (Supporting Information) show that the HOMO–LUMO gap in the CDs was 3.02 eV and ZIF‐8 was 4.13 eV, while the CDs@ZIF‐8 nanocomposites had narrower HOMO–LUMO gap of 2.22 eV (Zn─O) and 1.56 eV (Zn─N). The reduced bandgap of the CDs@ZIF‐8 nanocomposites could be attributed to the formation of Zn─O and Zn─N bonds caused by the combination of CDs and ZIF‐8, which facilitated more efficient electron and energy transfer, resulting in a low‐energy LUMO state.^[^ ^57^ ^]^ The reduced bandgap of the CDs@ZIF‐8 nanocomposites promoted photogenerated e^−^–h^+^ pairs separation and the generation of ^1^O_2_ and •O_2_^−^, thereby enhancing their photodynamic antimicrobial activity.^[^ ^58^ ^]^ Considering the key role of O_2_ in the photodynamic generation of ROS, the binding energies of O_2_ in ZIF‐8 and CDs@ZIF‐8 were calculated using DFT, which were ‐55.24 and ‐68.69 kJ mol^−1^, respectively (Table S4, Supporting Information). Therefore, CDs@ZIF‐8 had a higher binding affinity for O_2_ than ZIF‐8, contributing to the production of more ^1^O_2_ and •O_2_^−^. These findings show that the composite of CDs and ZIF‐8 could promote photo‐generated e^−^–h^+^ pairs separation, inhibit the recombination of photogenerated carriers, and enhance the binding affinity for O_2_, facilitating the generation of ^1^O_2_ and •O_2_^−^. Consequently, CDs@ZIF‐8 exhibited markedly enhanced photodynamic antimicrobial performance compared with CDs and ZIF‐8 alone.

### 2.6 Nanofibrous Films Structure Characterization

The CDs@ZIF‐8 nanocomposites exhibited dual photodynamic enhanced antimicrobial activity. Subsequently, to facilitate the application in the preservation of perishable fruits, PCL and EC were used as film‐forming substrates, and the MBS technology was employed to prepare the CDs@ZIF‐8/PCL/EC nanofibrous films with photodynamic antimicrobial activity. The nanofibrous films exhibited a smooth and even appearance, with the color deepening as CDs@ZIF‐8 nanocomposite contents increased (Figure S15A, Supporting Information). PCL/EC, 1%, 2.5%, and 5% CDs@ZIF‐8/PCL/EC nanofibrous films were found to be composed of uniform and smooth nanofibers with average fiber diameters of 342.05, 307.09, 324.28, and 290.04 nm, respectively (Figure S15B–E, Supporting Information). Notably, incorporating of CDs@ZIF‐8 nanocomposites did not compromise the smoothness of the nanofibers or induce significant changes in fiber diameter. The proportion of Zn was enhanced by increasing the CDs@ZIF‐8 nanocomposite content, confirming its successful incorporation into the PCL/EC nanofibrous films (Figures S16–S19, Supporting Information).

The FT‐IR spectrum of the different groups was broadly similar (Figure S20A, Supporting Information). The peaks at 3200–3600 cm⁻^1^ were assigned to ─OH stretching. The peaks at 2943 cm⁻^1^ and 2866 cm⁻^1^ were due to C─H stretching vibrations in the alkyl groups. All groups exhibited a peak at 1724 cm⁻^1^, owing to C═O stretching vibrations.^[^ ^26^ ^]^ Moreover, XRD was applied to determine the dispersion of CDs@ZIF‐8 nanocomposites in PCL/EC (Figure S20B, Supporting Information). PCL diffraction peaks were detected at 2*θ* = 21.4° and 23.8°. There were four characteristic peaks at 2*θ* = 7.4°, 12.8°, 16.5°, and 18.1°, which were the ZIF‐8 (011), (112), (013), and (222) crystal planes. Unique crystal planes of the composite nanoparticles were detected in the CDs@ZIF‐8/PCL/EC nanofibrous films, suggesting that the CDs@ZIF‐8 nanocomposites were successfully spun into them.

Hydrophobicity is a critical indicator for evaluating food packaging, especially for food packaging such as fruit and vegetables that need to be stored in an environment with high relative humidity. Hydrophobic packaging materials resist erosion in high‐humidity environments, preventing the structural collapse of the packaging. The water contact angle of the PCL/EC was relatively high at 131.55°, indicating that the PCL/EC was hydrophobic (Figure S21A, Supporting Information).^[^ ^59^ ^]^ Besides, the water contact angle increased with the concentration of CDs@ZIF‐8 nanocomposites (Figure S21B–E, Supporting Information), which could be attributed to the inherent superhydrophobicity of ZIF‐8.^[^ ^60^, ^61^ ^]^ Water vapor permeability is a crucial parameter for evaluating packaging materials. As shown in Figure S21F (Supporting Information), the water vapor permeability of all groups did not exhibit significant differences, suggesting that incorporating of CDs@ZIF‐8 nanocomposites did not notably impact the adsorption and effusion of water molecules across the nanofibrous films.

**Figure 6:**
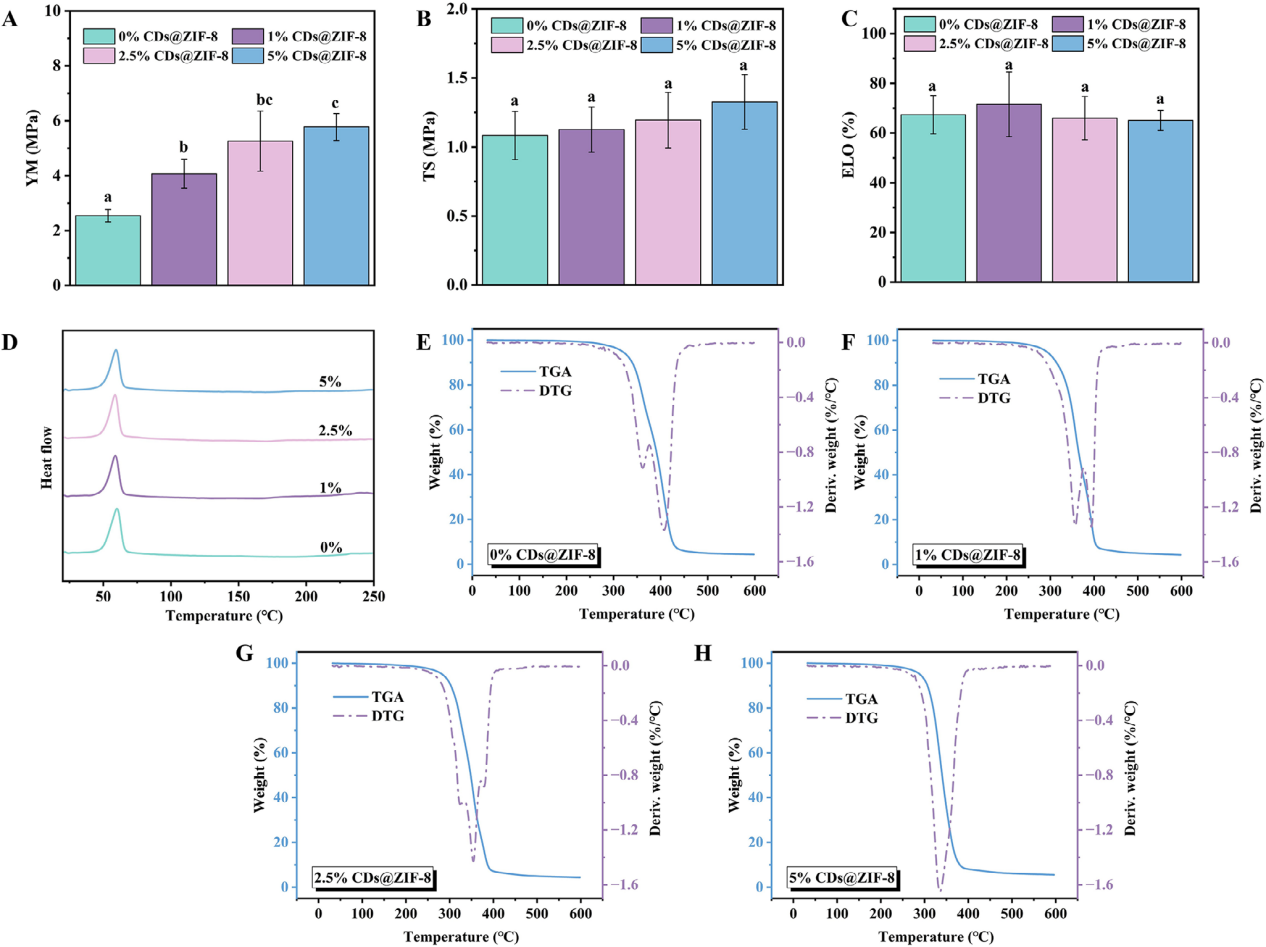
A) Young's modulus (YM), B) tensile strength (TS), and C) elongation at break (ELO) of the CDs@ZIF‐8/PCL/EC nanofibrous films (*n* = 3). D) DSC plot of the CDs@ZIF‐8/PCL/EC nanofibrous films. TGA and DTG analysis of the PCL/EC nanofibrous films containing E) 0%, F) 1%, G) 2.5%, and H) 5% (H) CDs@ZIF‐8 nanocomposites. Different letters (a–c) represent significant differences (*p < 0.05).

Good mechanical properties are essential for films, as they help to prevent product damage and thus maintain quality.^[^ ^62^, ^63^ ^]^ **Figure** **6**A shows that the Young's modulus was not significantly different between the groups. Figure 6B illustrates the tensile strength of the nanofibrous films, with the 5% CDs@ZIF‐8 group demonstrating superior performance. The tensile strength of the nanofibrous films increased with the proportion of CDs@ZIF‐8 nanocomposites, suggesting enhanced robustness. Elongation at break (ELO) indicates a material's ductility or flexibility. There were no significant differences in ELO between groups, suggesting that the CDs@ZIF‐8 nanocomposites did not affect the ductility of the nanofibrous films (Figure 6C). As depicted in Figure 6D and Table S5 (Supporting Information), all groups exhibited similar endothermic peaks and melting temperatures. The primary weight loss for the nanofibrous films occurred between 330 °C and 410 °C (Figure 6E–H), and the weight loss occurring at 280 °C–380 °C was associated with the sequential degradation of polysaccharides and polyesters.^[^ ^64^ ^]^

The safety of nanofiber food packaging materials is crucial for human health, and their cytotoxicity assessments have garnered widespread attention. The cytotoxicity of nanofibrous films was evaluated after CDs@ZIF‐8/PCL/EC nanofibrous films were exposed to visible light for 3 h and then co‐cultured with L929 cells for 24 h. The results show that the relative cell viability remained higher than 80% (Figure S22A, Supporting Information), confirming the low toxicity of the nanofibrous films. The results were further supported by live/dead cell imaging. Red staining, indicating dead cells, was barely visible. Green staining, indicating live cells, and most cells appeared green, indicating that most were alive (Figure S22B, Supporting Information). These results demonstrate that the CDs@ZIF‐8/PCL/EC nanofibrous films maintained high cell viability when co‐cultured with L929 cells, suggesting that the CDs@ZIF‐8/PCL/EC nanofibrous films possessed good biocompatibility and low toxicity.

In addition, the migration of Zn^2+^ from CDs@ZIF‐8/PCL/EC nanofibrous films was measured to evaluate the safety of the nanofibrous films. The results demonstrate a gradual increase in Zn^2+^ migration in the PBS solution over time, with a measured concentration of 2.09 mg L^−1^ at 12 d (Figure S23A, Supporting Information). Furthermore, the Zn^2+^ migration from the nanofibrous films to strawberries was 1.65 mg L^−1^ after 12 d storage (Figure S23B, Supporting Information). The European Plastics Regulation (EU) 2016/1416 (European Commission, 2016) has set a specific migration limit (SML) of 5 mg kg^−1^ for soluble zinc ions in food contact materials,^[^ ^65^ ^]^ and the U.S. National Institutes of Health has defined a daily intake of 40 mg d^−1^ for zinc in food contact materials.^[^ ^66^ ^]^ Notably, the Zn^2+^ migration observed in the present study (1.65 mg L^−1^ in strawberries and 2.09 mg L^−1^ in PBS solution after 12 d) was below these safety standards.

### 2.7 Antimicrobial Activity of Nanofibrous Films

**Figure 7:**
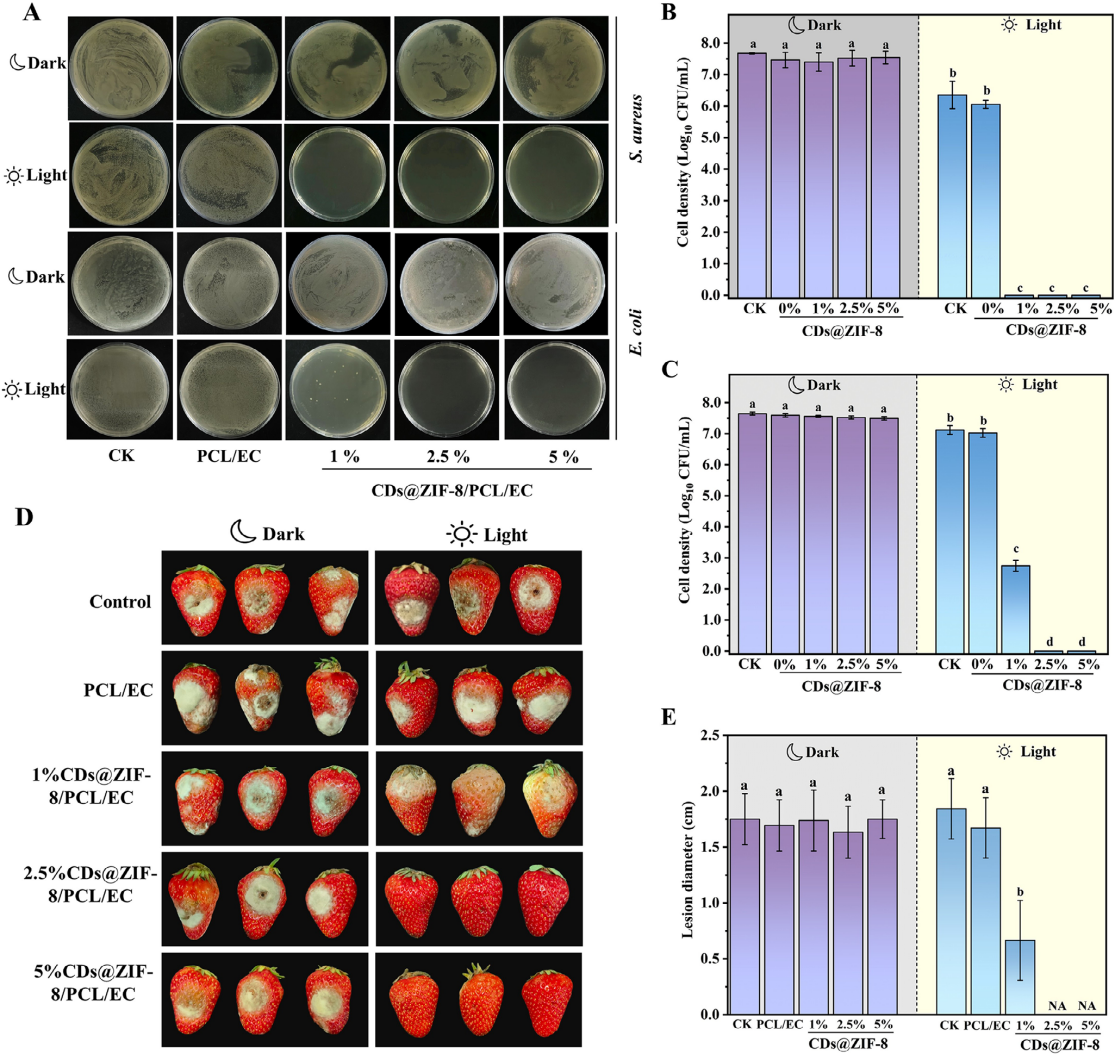
A) Antimicrobial performance of the CDs@ZIF‐8/PCL/EC nanofibrous films toward*S. aureus* and *E. coli*. Cell densities of B) *S. aureus* and C) *E. coli*. D) Inhibitory effect of CDs@ZIF‐8/PCL/EC nanofibrous films on inoculation of*B. cinerea* in strawberries in vivo under light and dark and E) the corresponding lesion diameters (*n* = 12). Different letters (a–d) represent significant differences (*p < 0.05).

The PCL/EC and CDs@ZIF‐8/PCL/EC nanofibrous films exhibited no significant antimicrobial properties under dark conditions (**Figure** **7**A), which was related to the CDs@ZIF‐8 nanocomposites having almost no ROS production under dark conditions. Remarkably, when exposed to visible light, the nanofibrous films containing 1% CDs@ZIF‐8 nanocomposites completely inhibited *S. aureus* growth, while those with 2.5% CDs@ZIF‐8 nanocomposites completely suppressed *E. coli* growth. Specifically, the nanofibrous films with 1% CDs@ZIF‐8 nanocomposites content could effectively eliminate 7.68 lg of *S. aureus* and 4.90 lg of *E. coli*, and the nanofibrous films with 2.5% CDs@ZIF‐8 nanocomposites content could effectively eliminate 7.64 lg of *E. coli* (Figure 7B,C). The results show that incorporating CDs@ZIF‐8 nanocomposites endowed the nanofibrous films with superior photodynamic antimicrobial properties.

Post‐harvest rot caused by fungal pathogens is a major contributor to fruit and vegetable losses.^[^ ^67^ ^]^ In the present study, strawberries, a representative perishable fruit, were selected and inoculated with a *B. cinerea* spore suspension to evaluate the in vivo antifungal activity of the nanofibrous films. Figure 7D shows that none of the groups showed significant antifungal activity under dark conditions. Moreover, the control and PCL/EC nanofibrous film groups showed no significant antifungal activity under light conditions (Figure 7E). However, the nanofibrous films containing 1%, 2.5% and 5% CDs@ZIF‐8 nanocomposites demonstrated significant antifungal activity compared with the control and PCL/EC nanofibrous film groups under light conditions. The nanofibrous films effectively delayed the spoilage of strawberries by inhibiting the growth of *B. cinerea*. In particular, the 2.5% and 5% CDs@ZIF‐8 nanocomposites nanofibrous films effectively eradicated *B. cinerea* spores, with strawberries remaining intact and clean, showing no fungal growth. Consequently, the 2.5% CDs@ZIF‐8 nanocomposites nanofibrous films were selected for subsequent strawberry storage preservation experiments.

### 2.8 Effect of Nanofibrous Films on Strawberry Preservation

**Figure 8:**
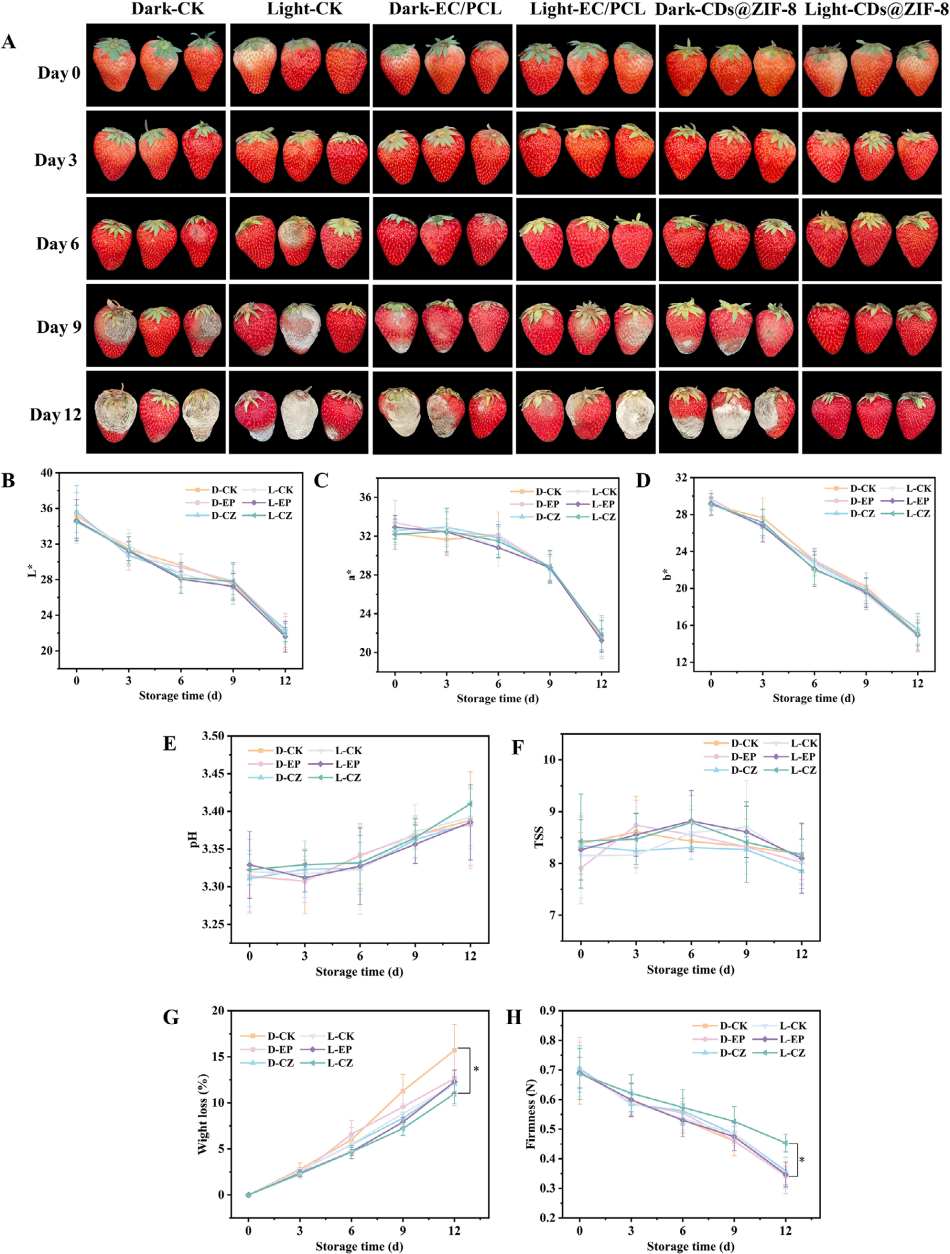
A) Morphological photographs of strawberries in different treatment groups. Changes in B) *L*, C) *a*, and D) *b* values of strawberries. Changes in E) pH, F) TSS, G) weight loss, and H) firmness of strawberries in different treatment groups (*n* = 12), *p < 0.05. D‐CK: control group under dark; L‐CK: control group under light; D‐EP: PCL/EC treatment group under dark; L‐EP: PCL/EC treatment group under light; D‐CZ: 2.5% CDs@ZIF‐8/PCL/EC treatment group under dark; L‐CZ: 2.5% CDs@ZIF‐8/PCL/EC treatment group under light.

The CDs@ZIF‐8/PCL/EC nanofibrous films demonstrated prominent photodynamic antimicrobial activity, good safety, and good physicochemical properties, showing great potential for strawberry preservation. This study assessed the films’ impact on the appearance quality of the strawberries. **Figure** **8**A shows no significant differences in the appearance of strawberries among the groups within the first 3 d. The strawberries exhibited signs of partial rot and spoilage in the control group at 6 d. Between 9 d and 12 d, the extent of rot and spoilage increased in strawberries from the control and PCL/EC groups, resulting in a loss of edibility. By contrast, after photodynamic treatment, the strawberries in the CDs@ZIF‐8/PCL/EC nanofibrous films group still maintained good quality without rot and spoilage at 12 d.

The quality indicators of external color, TSS, pH, weight loss rate and firmness, are important factors in consumers’ acceptance of fresh fruits.^[^ ^68^ ^]^ The color changes in the strawberries were analyzed with a colorimeter, and no significant differences were found among the treatment groups (Figure 8B–D). Similarly, pH and the soluble solids values of the strawberries had no significant differences under different treatments (Figure 8E,F), indicating that the nanofibrous films did not affect the strawberries’ appearance and nutritional quality. Figure 8G shows that the weight loss rate of the CDs@ZIF‐8/PCL/EC nanofibrous films under light group was significantly lower than that of the control group on 12 d (*p* < 0.05). The results were consistent with the findings of Zhao et al., who demonstrated that carbon nanodots/silk fibroin films could effectively reduce the weight loss of strawberries.^[^ ^69^ ^]^ Moreover, the firmness of the strawberries gradually declined as the storage time increased (Figure 8H), but the firmness decline of strawberries treated with the CDs@ZIF‐8/PCL/EC nanofibrous films group after the photodynamic treatment was the slowest. This was mainly because the CDs@ZIF‐8 nanocomposites in the nanofibrous films had dual photodynamic antimicrobial activity, which could reduce the damage of microorganisms to strawberries and make them have better peel strength. These results indicate that the CDs@ZIF‐8/PCL/EC nanofibrous films had good preservation effect, which could extend the shelf life of strawberries from 6 to 12 d after photodynamic treatment, which was better than some previously reported work on strawberry preservation materials (Table S6, Supporting Information). Moreover, our results show that after photodynamic antimicrobial treatment based on CDs@ZIF‐8/PCL/EC nanofibrous films, the strawberries were still well preserved during subsequent long‐term dark storage. Indeed, previous studies also indicate that photodynamic antimicrobial materials were able to maintain the long‐term preservation effect under dark conditions after photodynamic treatment.^[^ ^70^, ^71^ ^]^ Therefore, considering the limitation of weak effect in dark environments of CDs@ZIF‐8/PCL/EC nanofibrous films, it is suggested that the photodynamic treatment should be carried out before storage, which could not only achieve green efficient sterilization, but also provide a good preservation effect under subsequent dark storage conditions. Overall, the CDs@ZIF‐8/PCL/EC nanofibrous films had good preservation effect of strawberries and are expected to replace traditional chemical preservatives to realize the green antimicrobial and preservation of strawberries.

## 3 Conclusions

In the present study, CDs@ZIF‐8/PCL/EC nanofibrous films were prepared for the preservation of perishable fruits, and the synergistically enhanced antimicrobial mechanism of CDs and ZIF‐8 was elucidated by optoelectronic property analysis and DFT calculations. The results show that the CDs@ZIF‐8 nanocomposites displayed synergistically enhanced photodynamic antimicrobial properties for *E. coli* (7.63 lg elimination) and *S. aureus* (7.27 lg elimination), significantly outperforming CDs or ZIF‐8 alone. The enhanced photodynamic antimicrobial activities between CDs and ZIF‐8 could be attributed to two synergistic ways. On one hand, ZIF‐8 prevented CDs’ self‐aggregation, and CDs enhanced ZIF‐8′s light absorption. On the other hand, binding CDs and ZIF‐8 reduced the bandgap, promoted photo‐generated e^−^–h^+^ pairs separation, and improved the binding affinity for O_2_, resulting in increased •O_2_^−^ and ^1^O_2_ production. Subsequently, the CDs@ZIF‐8 nanocomposites were uniformly incorporated into PCL/EC using MBS to prepare CDs@ZIF‐8/PCL/EC nanofibrous films with superior photodynamic antimicrobial activity. The CDs@ZIF‐8/PCL/EC nanofibrous films also demonstrated low cytotoxicity and their Zn^2^⁺ migration was below both European and American safety standards. Furthermore, these nanofibrous films extended the shelf life of strawberries from 6 to 12 d. Our research presents a novel CDs‐MOF hybrid strategy that significantly enhances photodynamic antimicrobial efficacy while achieving safe and efficient preservation of perishable fruits.

## 4 Experimental Section

### Chemicals

Urea, citric acid, 2‐methylimidazole, zinc acetate, methanol, imidazole, 1,3‐diphenylisobenzofuran, p‐nitrosodimethylaniline, nitro‐blue tetrazolium, and 2,2,2‐trifluoroethanol were obtained from Macklin Co., Ltd. (Shanghai, China). Polycaprolactone and ethyl cellulose were purchased from Aladdin Co., Ltd. (Shanghai, China).

### Synthesis of CDs@ZIF‐8 Nanocomposites

CDs@ZIF‐8 nanoparticles were prepared using coordination self‐assembly. The CDs addition content was determined in the pre‐experiment. The results show that the CDs addition of 100 mg had the highest yield and good antimicrobial ability and therefore was selected for the present study to synthesize CDs@ZIF‐8 nanocomposites. Specifically, CDs (100 mg) were dispersed in methanol (40 mL) and blended with zinc acetate (10 mL). The mixture was quickly injected into 2MeIM (40 mL) and vigorously stirred. Then, the CDs@ZIF‐8 nanoparticles were collected via centrifuging and drying.

### Antimicrobial Experiment

The antimicrobial effects of the prepared samples were assessed on *E. coli*, *S. aureus* and *B. cinerea*. Bacterial and *B. cinerea* spore suspensions were spiked with the prepared samples. Sterile saline was used instead of the material in the control group. The mixture was exposed to visible light (450 nm, 60 mW cm^−2^) to explore the photodynamic antimicrobial effect of the prepared samples with different light exposure times. The suspensions (100 µL) of *E. coli* and *S. aureus* were uniformly distributed on plates after exposure to light for 0, 30, 60, 120 and 180 min. Then, various doses of CDs@ZIF‐8 composites were mixed with bacterial and *B. cinerea* spore suspensions. After a period of light exposure, the suspensions (100 µL) were spread uniformly on the culture medium to observe the growth of bacteria and fungi.

### Detection of ROS

The reactive oxygen species generated by the samples were determined using a UV–visible spectrophotometer (UV‐2600, Shimadzu, Japan) after a photodynamic treatment (450 nm, 60 mW cm^−2^) and a dark treatment.^[^ ^43^, ^44^, ^45^ ^]^ First, the •O_2_^−^ generation was detected using the colorimetric reaction of NBT. Briefly, the prepared samples (400 µg mL^−1^) were added to NBT (100 µg mL^−1^), and absorption at 259 nm was recorded at 15 min intervals. Subsequently, the generation of ^1^O_2_ was detected by RNO. The mixture of RNO, imidazole, and the prepared samples (400 µg mL^−1^) was recorded every 15 min at an absorbance of 440 nm. Then, ^1^O_2_ production was assessed with a DPBF fluorescent probe. The prepared samples (400 µg mL^−1^) were mixed with DPBF (20 µmol L^−1^, DMSO as solvent), and absorption at 416 nm was determined every 10 s. Pure NBT, RNO, and DPBF solutions without the prepared samples were used as controls.

### Intracellular ROS Determination

The bacteria suspension was incubated with the prepared samples. The control group was a light and dark treatment group with no sample. The red fluorescent dye DHE was then mixed with the cell suspension and incubated for 60 min. After centrifugation, the staining solution was discarded from the mixture. Afterward, the fluorescence intensity of ROS could be detected using a confocal laser scanning microscope.

### Preparation of Nanofibrous Films

The preparation method was based on the previous experimental method by the team, and was shown below.^[^ ^28^ ^]^ The nanofibrous films were prepared using the MBS machine (JNS‐MBS‐01, Janus New‐Materials Co., Ltd, Nanjing, China). PCL particles of 0.65 g; EC powder of 0.35 g; and CDs@ZIF‐8 nanocomposites of 0, 0.1, 0.25, and 0.5 g were placed in 10 mL of trifluoroethanol and mixed uniformly to prepare a spinning solution. The nanofibers obtained were designated as PCL/EC nanofibrous films, 1% CDs@ZIF‐8/PCL/EC nanofibrous films, 2.5% CDs@ZIF‐8/PCL/EC nanofibrous films, and 5% CDs@ZIF‐8/PCL/EC nanofibrous films.

### Strawberry Preservation Experiment

“Hongyan” strawberries of similar sizes and maturity and without damage or disease were selected and then divided into six groups: control group under light, control group under dark, PCL/EC treatment group under light, PCL/EC treatment group under dark, 2.5% CDs@ZIF‐8/PCL/EC treatment group under light, and 2.5% CDs@ZIF‐8/PCL/EC treatment group under dark. The strawberries were exposed to dark and visible light (450 nm, 60 mW cm^−2^) for 3 h and then stored at 10 °C and 90% RH for 12 d. The physiological quality of the strawberries was measured at 0, 3, 6, 9, and 12 d.

The strawberry color changes were measured using a colorimeter (MiniScan XE Plus, HunterLab, Virginia). The TSS and pH levels were determined using a portable hand‐held sugar meter (PR‐101α, ATAGO, Japan) and a pH meter (PB‐10, Sartorius, Germany). Strawberry firmness was assessed by a texture analyzer (TA‐XT2i, Stable Micro Systems, UK). The weight loss rate (Δ*m*) (%) of strawberries was determined with the following Equation (1):

(1) $$\Delta m=\frac{m_{0}-m_{1}}{m_{0}}\;\times 100\%$$

where *m*_1_ is the current weight of the fruit, and *m*_0_ is the original weight.

### Statistical Analysis

Statistical analysis was performed using three independent replicates. Data were expressed as mean ± standard deviation (SD). One‐way analysis of variance (ANOVA) was conducted using SPSS (version 27.0, IBM, USA), with significance at *p* < 0.05.

## Conflict of Interest

The authors declare no conflict of interest.

## Supporting information



Supporting Information

## Data Availability

The data that support the findings of this study are available from the corresponding author upon reasonable request.
