# Supplementary material for: Fabrication of Dual Photodynamic Enhanced Antimicrobial CDs@ZIF‐8/Polycaprolactone/Ethyl Cellulose Nanofibrous Films for Fruit Preservation
Source: Adv Sci (Weinh). 2025 Jun 23;12(35):e03567. doi: 10.1002/advs.202503567 (PMC12462961; doi:10.1002/advs.202503567)
Supplement: Supplementary file 1 — Supporting Information [file ADVS-12-e03567-s001.docx]

**Supporting Information**

**Fabrication of Dual Photodynamic Enhanced Antimicrobial** **CDs@ZIF-8/Polycaprolactone/Ethyl Cellulose Nanofibrous Films for Fruit Preservation**

*Yongrui Ti, Ziqiang Qin, Xiaohong Guo, Zheng Wu, Qi Jiang, Beihua Dong, Olaniyi Amos Fawole,* *Di Wu ^*^, Su Chen, Xian Li, Kunsong Chen*

Y. Ti, Z. Qin, Z. Wu, D. Wu, X. Li, K. Chen

College of Agriculture & Biotechnology/Zhejiang Key Laboratory of Horticultural Crop Quality Improvement/Key Laboratory of Ministry of Agriculture and Rural Affairs of Biology and Genetic Improvement of Horticultural Crops (Growth and Development), Zhejiang University, Hangzhou 310058, P. R. China.

X. Guo, D. Wu

College of Biosystems Engineering and Food Science, Zhejiang University, Hangzhou 310058, P. R. China.

Q. Jiang, B. Dong

Hualian Machinery Group Co., Ltd, Wenzhou, 325035, P. R. China.

O. A. Fawole

Postharvest Research Laboratory, Department of Botany and Plant Biotechnology, University of Johannesburg, P.O. Box 524, Auckland Park, 2006 Johannesburg, South Africa.

D. Wu

Zhejiang University Zhongyuan Institute, Zhengzhou 450000, P. R. China.

S. Chen

State Key Laboratory of Materials-Oriented Chemical Engineering & College of Chemical Engineering, Jiangsu Key Laboratory of Fine Chemicals and Functional Polymer Materials, Nanjing Tech University, Nanjing, 210009, P. R. China.

*** *Corresponding author: Di Wu.* *Add/Affiliation: 866 Yuhangtang Road. Hangzhou, 310058, China. Tel: 0086-571-88982226. Email: di_wu@zju.edu.cn (Di Wu)*

**This** **file includes:**

Supplementary experimental methods

Figure S1 to S23

Table S1 to S6

References

**Supplementary experimental methods**

*Synthesis of CDs nanomaterials*: Carbon dots were prepared through the hydrothermal method.^[1]^ Briefly, urea (0.3 g) and citric acid (0.96 g) were mixed in 10 mL of deionized water. The mixture was transferred to the hydrothermal kettle, where it underwent heating at 160°C for 2 h. The reaction solution was then collected and filtrated using a 0.22-μm microporous filter. The carbon dots were dialyzed for 48 h using a dialysis bag with a molecular weight of 1000 Da. After that, the dialysate was collected and dried to obtain the CDs.

*Synthesis of ZIF-8*: The preparation of ZIF-8 was conducted following earlier studies, with some adjustments.^[2]^ Briefly, 0.36 g of Zn(CH_3_COO)_2_ and 11.35 g of 2-MeIM were dissolved in 50 mL of methanol. The reaction solution was stirred thoroughly for 30 min. ZIF-8 was obtained by collecting the resulting solid through centrifugation, washing it three times, and then drying it.

*Synthesis of CDs/ZIF-8*: The CDs/ZIF-8 nanocomposites were synthesized by a simple mechanical mixing method. The aqueous solution of CDs was mixed with the pre-synthesized ZIF-8 powder with the same content of CDs, and the powder was stirred vigorously for 2 h at room temperature. Then, the CDs/ZIF-8 nanoparticles were collected by centrifugation and drying.

*Structural characterization*: TEM images of CDs, ZIF-8, and CDs@ZIF-8 were obtained using a transmission electron microscope (2100F, JEOL JEM, Japan) at an accelerating voltage of 120 kV. SEM images of the as-prepared samples were obtained at an accelerating voltage of 3 k V, and EDX images of the as-prepared samples were obtained at an accelerating voltage of 15 k V using a scanning electron microscope (Sigma 300, ZEISS, Germany) equipped with an energy dispersive X-ray spectrometer. XRD patterns of CDs, ZIF-8, and CDs@ZIF-8 were obtained on an X-ray diffractometer (XRD) (SmartLab SE, Rigaku, Japan). The fluorescence properties of the CDs were determined by the ultraviolet light at 365 nm. X-ray photoelectron spectroscopy (XPS) (K-Alpha, Thermo Scientific, Massachusetts) was performed on a spectrometer equipped with a monochromatic Al K α X-ray source (1486.6 eV) with a spot size of 400 μm, an operating voltage of 12 kV, and a filament current of 6 mA. The samples were analyzed in vacuum (P < 10^-7^ mbar) at a pass energy of 150 eV (full spectrum scan, step size of 1 eV) and 50 eV (high resolution scan, step size of 0.1 eV), and all peaks were charge-corrected using the C1s binding energy of 284.80 eV as the energy standard. Fourier transform infrared spectra (FT-IR) of the samples were measured in the range of 400-4000 cm^-1^ with a resolution of 4 cm^-1^ using a Fourier transform infrared spectrometer (Nicolet iS20, Thermo Fisher Scientific, Massachusetts). The zeta potential of CDs, ZIF-8 and CDs@ZIF-8 samples was measured using a nanometer (Nano ZS90, Malvern Zetasizer, UK). The Brunauer-Emmett-Teller (BET) surface area and pore structure of were measured by surface area and porosity analyzer (Autosorb IQ3, Quantachrome, USA). The photoluminescence emission spectra of CDs, ZIF-8 and CDs@ZIF-8 were recorded using a fluorescence spectrophotometer (FLS100, Edinburgh, UK).

*Photostability test*: The CDs@ZIF-8 nanocomposites (tested at a concentration of 400 μg/mL for *E. coli* and 100 μg/mL for *S. aureus*) were first exposed to continuous visible light for 5-30 h and then co-cultured with the corresponding bacteria under visible light (3 h for *E. coli* and 2 h for *S. aureus*). Subsequently, the bacteria suspensions (100 μL) were dispersed uniformly on solid culture medium to observe the growth of colonies.

*Microstructure observation*: The morphologies of *E. coli* and *S. aureus* were observed by TEM (H7650, Hitachi, Japan) when exposed to light and dark. The sample-treated bacterial suspension was centrifuged and placed in 2.5% glutaraldehyde for overnight fixation. After that, the sample was fixed again in 1% osmium acid for 1.5 h. Subsequently, the osmium acid was rinsed with PBS buffer, and the sample was dehydrated with different concentration ethanol solutions and then dehydrated with gradient concentrations (90% and 95%) of acetone solution. The samples were infiltrated with resin, dried, sectioned, stained, and observed by TEM.

*Photoelectrochemical measurements*: To explore the optical and electrochemical properties of the constructed CDs, ZIF-8 and CDs@ZIF-8 nanomaterials, UV-vis diffuse reflectance spectroscopy (DRS), ultrafast transient absorption (TA) spectroscopy, Mott-Schottky, electrochemical impedance spectroscopy (EIS), and transient photocurrent responses were measured. UV-visible diffuse reflectance spectra (UV-vis DRS) were recorded on a UV-visible spectrophotometer (UV-3600 Plus, Shimadzu, Japan), using BaSO_4_ as the reference standard. Transient absorption (TA) spectra of samples were obtained using a pump-probe transient absorption spectroscopy measurement system (LP920, Edinburgh Instruments, UK). All samples in measurements were in solution and obtained with 1mm quartz cuvettes. The pump beam was acquired by the fundamental output from Yb: KGW laser (1030 nm, 100 kHz, Light Conversion Ltd) was separated into multiple light beams. The temporal delay between them is controlled via a motorized delay stage. The pump and probe pulses overlapped spatially in the sample and the probe light was collected by a linear array detector (TA-100, Time-Tech Spectra, LLC). Electrochemical tests were performed on a CHI 760E electrochemical system equipped with a standard three-electrode electrochemical cell. 10 mg CDs, ZIF-8 and CDs@ZIF-8 powder samples were dispersed in 1 mL water/ethanol solution, followed by 50 μL Nafion solution, which was then ultrasonic for 30 min to form a homogeneous suspension. 100 μL of the suspension was dropped on ITO glass and dried at room temperature for photoelectric testing. An Ag/AgCl electrode was applied as the reference electrode, and platinum wire as the counter electrode. 0.2 mol L^-1^ Na_2_SO_4_ was used as the electrolyte. The Mott-Schottky plot was performed at a fixed frequency of 2000 Hz. The EIS measurement was conducted at a polarized potential of 10 mV with a frequency range of 100 kHz to 0.1Hz. The transient photocurrent response of the electrode to the light on/off period was performed with a light on/off interval of 20 s and six light on/off cycles.

*Density functional theory calculations*: Density functional theory (DFT) calculations were performed using the Dmol3 code in Materials Studio 2023 software. The more reliable Generalized-Gradient-Approximation (GGA) function was selected as the exchange-correlation potential type of DFT calculation. The Perdew-Burke-Ernzerhof (PBE) function was selected as the gradient-corrected functional. After the molecular model was established, the geometry was first optimized to achieve a stable geometry and then the energy calculations were performed. The overall quality for the DMol3 calculation was set to Fine. The energy of the highest occupied molecular orbital (HOMO) and the lowest unoccupied molecular orbital (LUMO) were calculated using “Orbitals selection”, and the resolution of the grid was 0.25 Å. The electron density and electrostatic potential of the molecule were calculated by “Electron density selection” and “Electrostatics selection”. “Population analysis” was used to calculate the total Mulliken charge on each atom and the charge transfer between molecules. The integration accuracy of the numerical integration of the Hamiltonian was set to Fine, and the self-consistent field (SCF) tolerance was set to 10^-6^.

*Characterization of the nanofibrous films*: After the nanofibrous films was gold-sprayed, the SEM images of the nanofibers were obtained at an accelerating voltage of 3 kV using a scanning electron microscope (Sigma 300, ZEISS, Germany) equipped with an energy dispersive X-ray spectrometer, and the EDX images of the nanofibers were obtained at an accelerating voltage of 15 kV. The SEM images of multiple regions of the nanofibrous films were analyzed using Nano Measurer 1.2 software, and 100 nanofibers were randomly selected from different SEM images for diameter measurement, and the average value was calculated. The samples were scanned using an X-ray diffractometer (Ultima IV, Rigaku, Japan) (scanning angle (2θ) from 5° to 90°, scanning rate of 2° min^-1^), and the XRD patterns of each group of nanofibrous films were obtained. Fourier transform infrared spectrometer (Nicolet iS20, Thermo Scientific, Massachusetts) was used for measurement. About 3 mg of each group of nanofibrous films samples were prepared and ground into powder with potassium bromide. The FT-IR spectra of the nanofibrous films were measured at a resolution of 4 cm^-1^ in the wavelength range of 4000-400 cm^-1^. The water contact angle (WCA) of the nanofibrous films was measured using a video contact angle tester (SDC 350KS, Kunshan Shengding Testing Instrument Co., Ltd., China). After the sample was placed flat on the test platform, about 3.5 μL of distilled water was dropped on the film surface using the automatic titration system of the equipment and a photo was taken. The contact angle was measured by the height method. The water vapor transmission rate (WVTR) of each group of nanofibrous films was tested using the cup method according to our previous method.^[3]^ Assemble the test container: Add 10 mL of distilled water to the permeation cup, place the pre-cut film sample close to and completely cover the cup mouth, and then fix the sample with a screw and a hollow ring clamp with an inner diameter equal to the diameter of the permeation cup mouth to create an environment with a humidity of 100% RH in the permeation cup. Place the assembled container in a desiccator filled with dry silica gel (0% RH) at ambient temperature, and weigh the permeation cup after 1 h of equilibration, and then weigh it every 12 h. The film thickness is the average value of the three symmetrical positions, and then the WVTR is calculated using formula (1):

|  | $\text{WVTR}\text{=}\frac{\text{ΔM}\text{×}\text{d}}{\text{Δt}\text{×}\text{A}\text{×}\text{Δp}}$ | （1） |
| --- | --- | --- |

where *ΔM* is the weight difference (g), *d* is the thickness of the film (mm), *Δt* is the measurement interval (h), *A* is the area of the cup mouth (9.621 × 10^-4^ m^2^), and *Δp* is the water vapor pressure difference between the inside and outside of the film (3.1671 kPa at ambient temperature).

Thermogravimetric and differential thermal analysis of the nanofibrous films were performed using a synchronous thermal analyzer (TG-DSC) (STA 449 F3, Netzsch, Germany). About 30 mg of the sample was placed in a crucible and heated from 30°C to 600°C at a heating rate of 10°C/min under a nitrogen atmosphere to obtain the scanning curve of each sample in this temperature range. The tensile properties of the nanofibrous films were measured at room temperature by an electronic universal testing machine (INSTRON 5982, Instron, Massachusetts). Each group of nanofibrous films was pre-cut into strips of 4 × 0.5 cm. The working load of the testing machine was set to 10 N, and then the sample was stretched at a moving rate of 10 mm/min until it broke, thereby obtaining the stress-strain curve of the nanofibrous films, and finally calculating its corresponding Young’s modulus, tensile strength and elongation at break.

*The cytotoxicity of the nanofibrous films*: Cytotoxicity was tested using the method described by Shen et al.^[4]^ Specifically, 10 mg of nanofibrous films exposed to visible light (450 nm, 60 mW/cm^2^) for 3 h were added to 10 mL of culture medium, and the supernatant was filtered through a 0.22-μm nanofibrous films to obtain an extraction solution. Human foreskin fibroblasts (L929) in the logarithmic growth phase were cultured in a 96-well plate, with approximately 6×10^3^ cells in each well, and cultured at 37°C, 5% CO_2_, and 90% RH until the cells adhered to the wall. Then, 100 μL of sample extracts at different concentrations (25, 50, 100, 200, and 400 μg/mL) were added to each well and incubated for 24 h. Next, 100 μL of 0.5 mg/mL MTT solution was added to each well and incubated for another 4 hours. Then, 100 μL of dimethyl sulfoxide was added to each well, and its absorption peak was recorded at 570 nm after gentle shaking for 10 min using an UV-visible spectrometer (UV-2600, Shimadzu, Japan). In addition, a live/dead assay was performed to further study the biocompatibility of the samples. Specifically, the cell suspension after co-culture with the sample extract was mixed with the dye solution and incubated at 37°C for 15 min, and the results were observed using a fluorescence microscope.

*Zn^2+^ migration test*: The release of Zn^2+^ from CDs@ZIF-8/PCL/EC nanofibrous films (200 mg) immersed in PBS solution (10 mL, pH=7.4) were evaluated at intervals of 3, 6, 9, and 12 d. Additionally, Zn^2+^ migration in strawberries that were wrapped with the CDs@ZIF-8/PCL/EC nanofibrous films, exposed to light for 3 h, and subsequently stored at 10℃ for 12 d were measured. Zn^2+^ levels were measured using the Zinc Colorimetric Assay Kit (Elabscience, E-BC-K137-M).

*Antimicrobial activity of nanofibrous films*: The CDs@ZIF-8/PCL/EC nanofibers with 0%, 1%, 2.5%, and 5% CDs@ZIF-8 nanocomposites (200 mg) added to *E. coli* and *S. aureus* suspensions (10 mL, 10^7^ CFU·mL^-1^) and incubated under darkness and light (450 nm, 60 mW/cm^2^) for 3 h. The bacterial suspension containing without nanofibers served as a control. Then, the mixed solutions (100 μL) were evenly spread on plates, and the counts of CFU were determined by colony counting. To investigate the antifungal activity of the nanofibrous films, ‘Hongyan’ strawberries were selected as a test object for the in vivo antifungal properties of the nanofibrous films. The 90% ripe ‘Hongyan’ strawberries were picked on the same day from an orchard in Zhejiang, China. *B. cinerea* was inoculated by the method of Wu et al. ^[5]^ A sterile inoculation needle was used to make a wound in the equatorial plane of the strawberry, and then a suspension of *B. cinerea* spores (5 μL, 10^6^ CFU·mL^-1^) was inoculated into the wound and covered with nanofibrous films. Afterwards, the strawberries covered with nanofibrous films were treated in the dark and visible light (450 nm, 60 mW/cm^2^) for 3 h and then cultured for 3 days (26°C, 75% RH) and the decay of the fruit was observed.

**Supplementary Figures:**


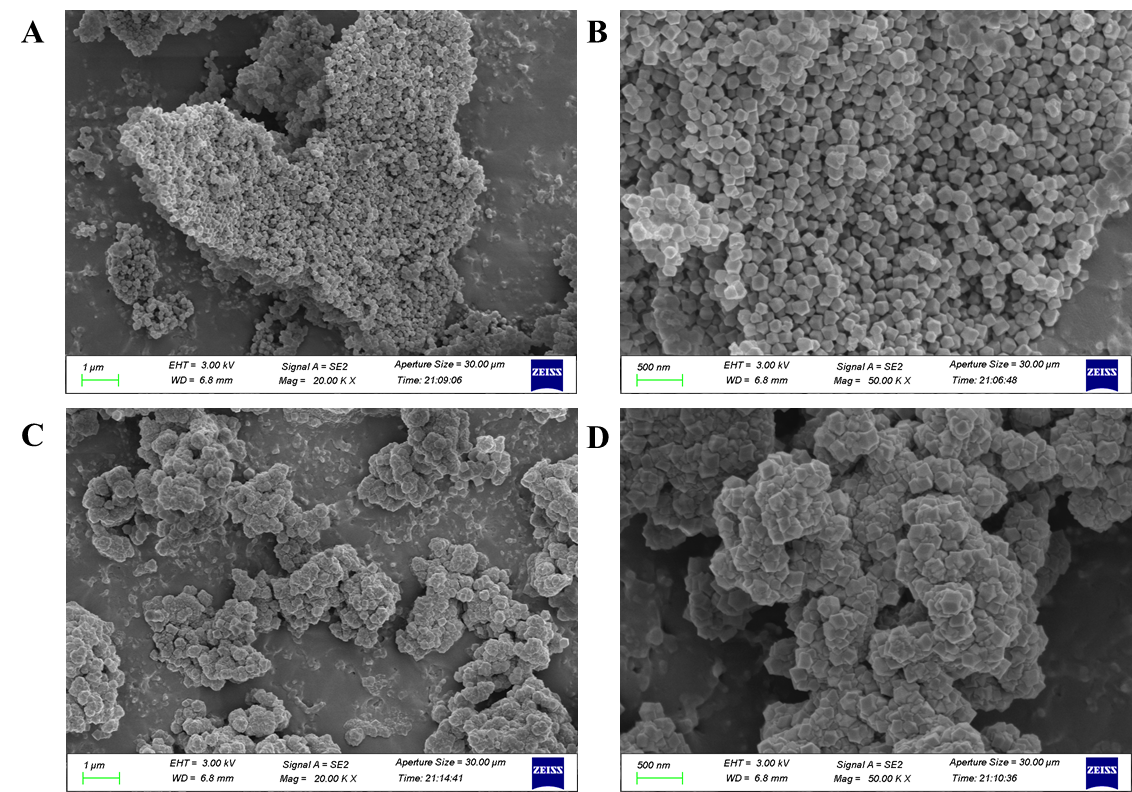


**Figure S1.** SEM images of ZIF-8 at A)1 μm and B) 500 nm, respectively. SEM images of CDs@ZIF-8 at C)1 μm and D) 500 nm, respectively.


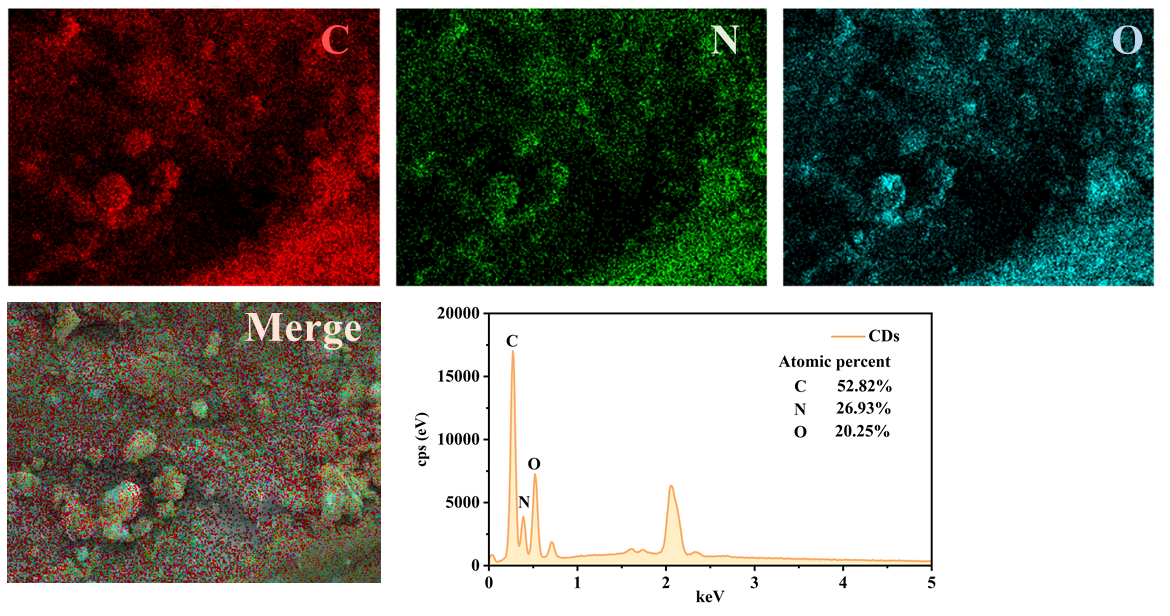


**Figure S2.** EDX spectroscopy elemental mapping images of CDs.


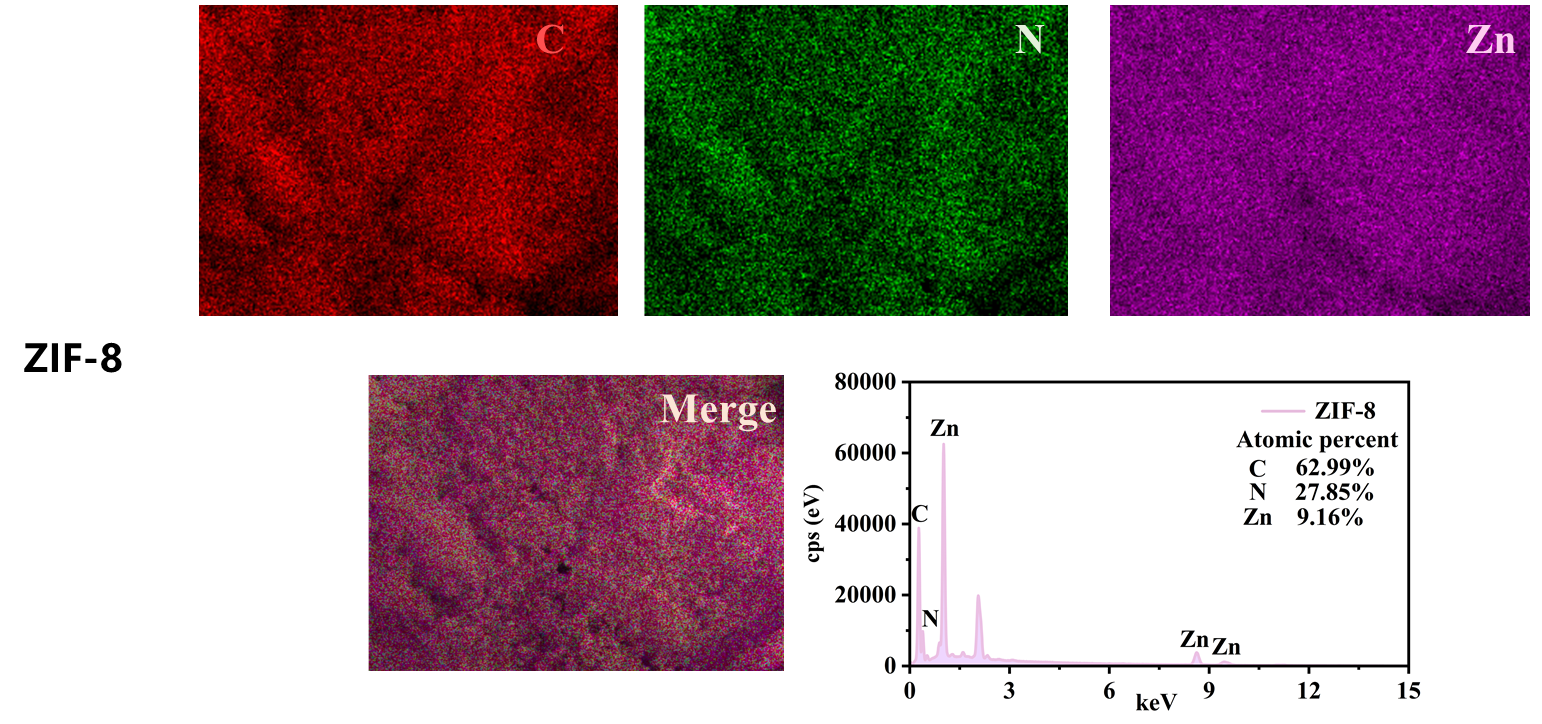


**Figure S3.** EDX spectroscopy elemental mapping images of ZIF-8.


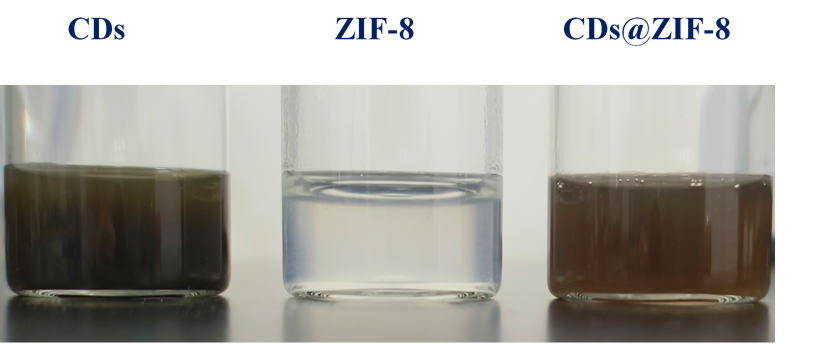


**Figure S4.** Visual appearance of CDs, ZIF-8 and CDs@ZIF-8 solutions.


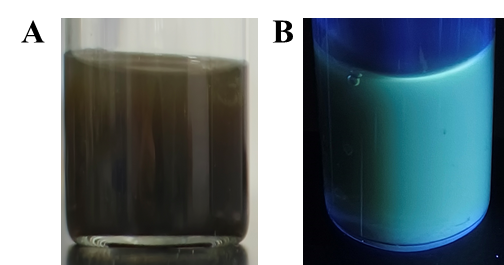


**Figure S5.** A)Optical images of CDs solution without UV light irradiation. B) CDs solution emitting fluorescence under UV light irradiation (λ ex = 365 nm).


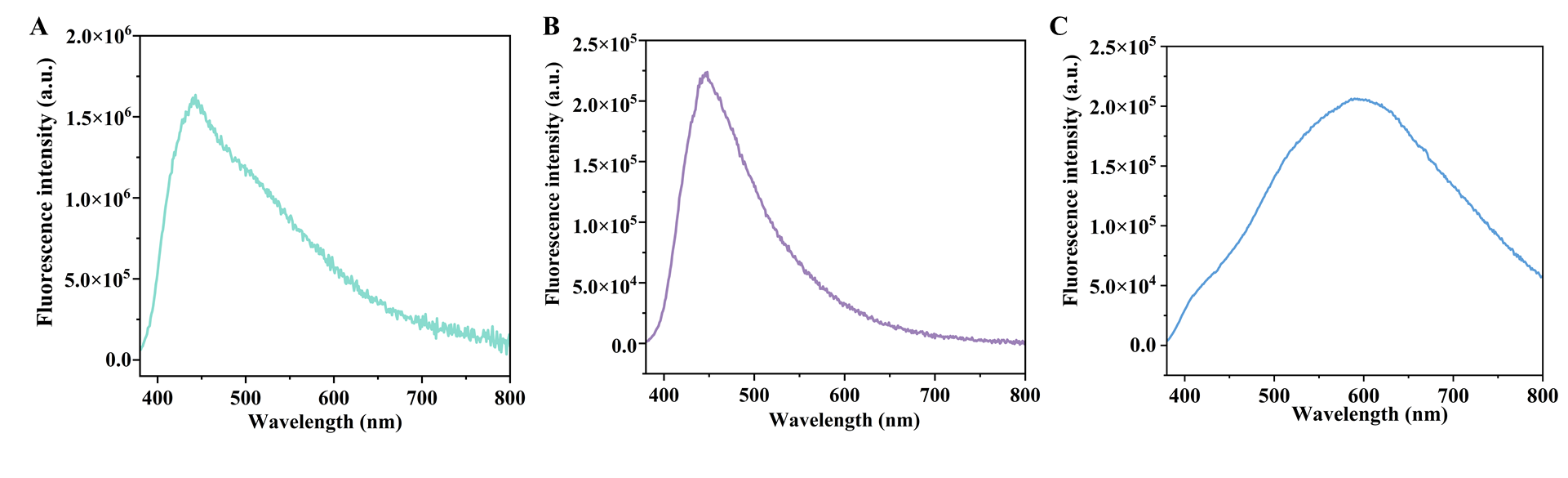


**Figure S6.** Fluorescence spectra of A) CDs, B) ZIF-8, and C) CDs@ZIF-8.


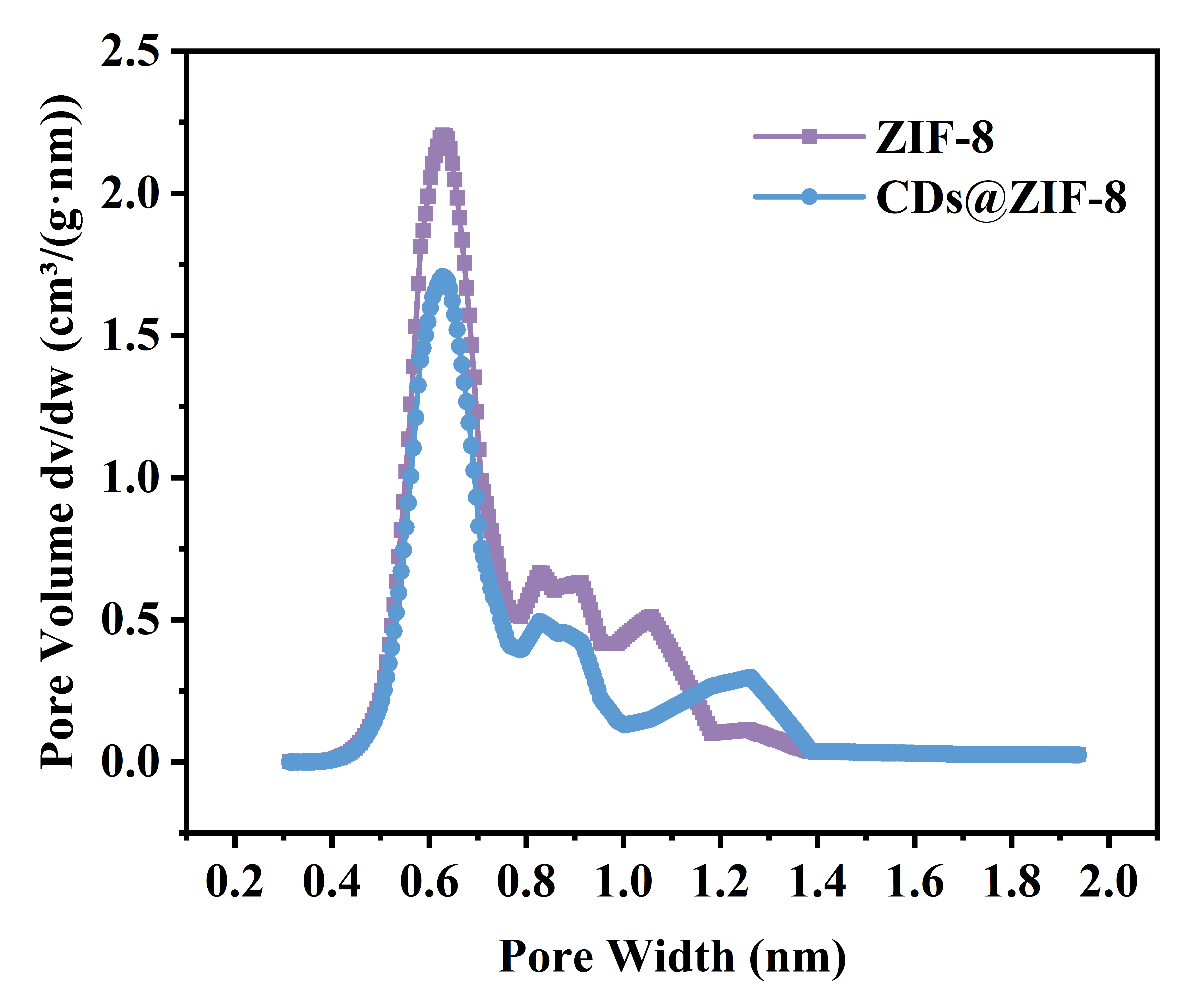


**Figure S7.** Pore width distribution curves of ZIF-8 and CD@ZIF-8 nanocomposites.


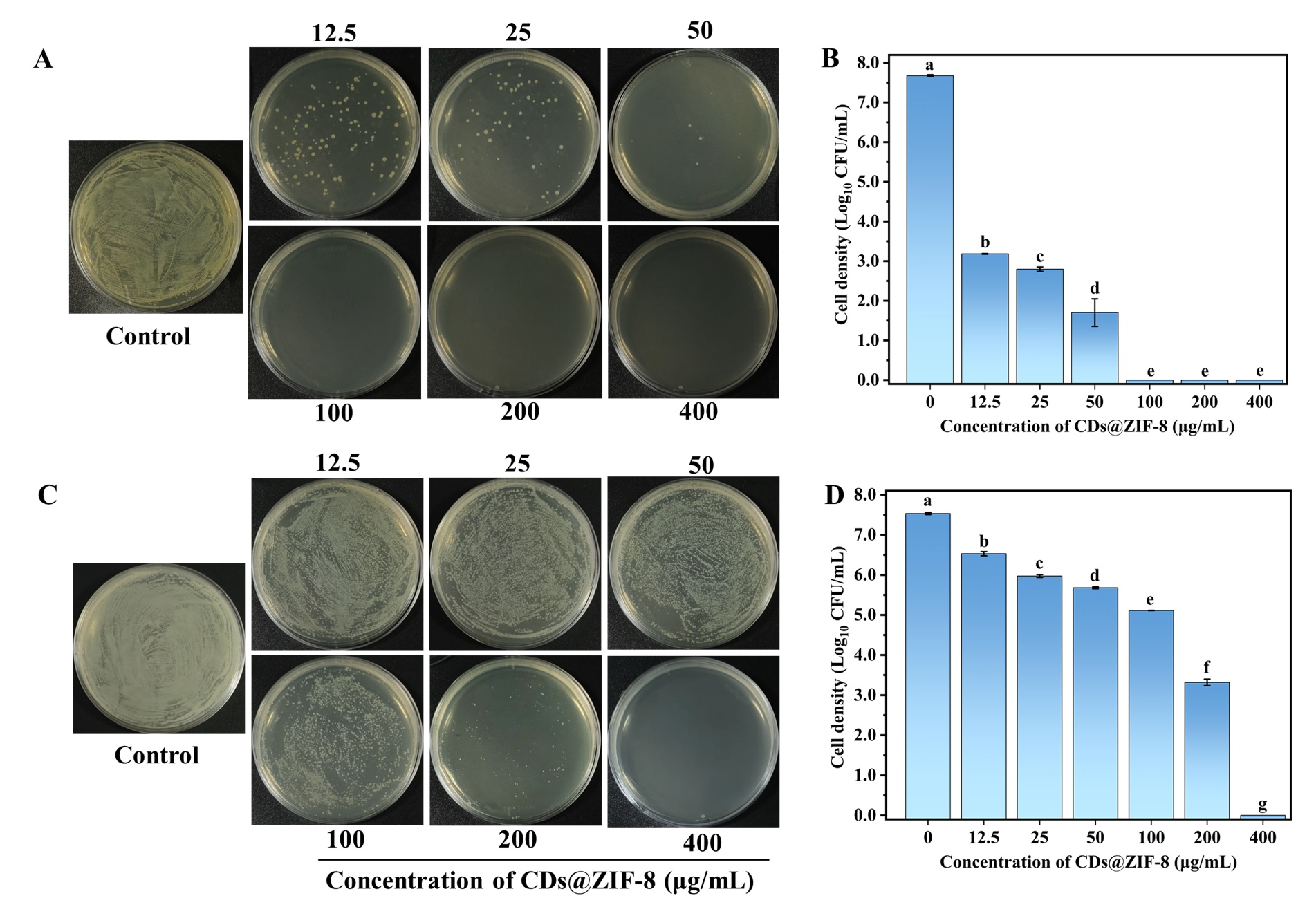


**Figure S8.** A) *S. aureus* and B) *E. coli* colony growth with different concentrations of CDs@ZIF-8 nanocomposites. Plots of changes of C) *S. aureus* and D) *E. coli* with different concentrations (n=3 in each group). Different letters (a-g) represent significant differences (**p* < 0.05).


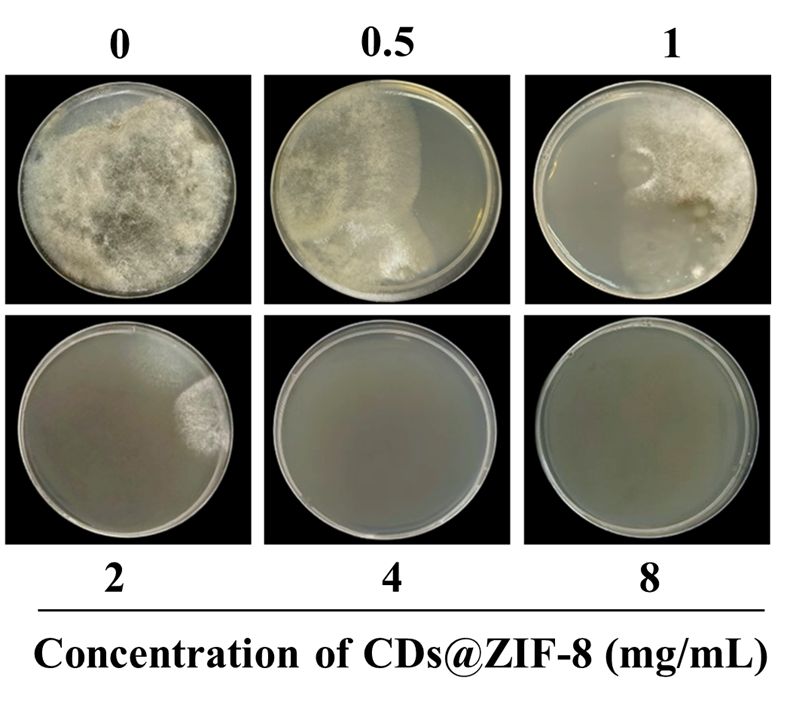


**Figure S9.** The mycelium growth of *Botrytis cinerea* was treated with different concentrations of CDs@ZIF-8 nanocomposites under light.


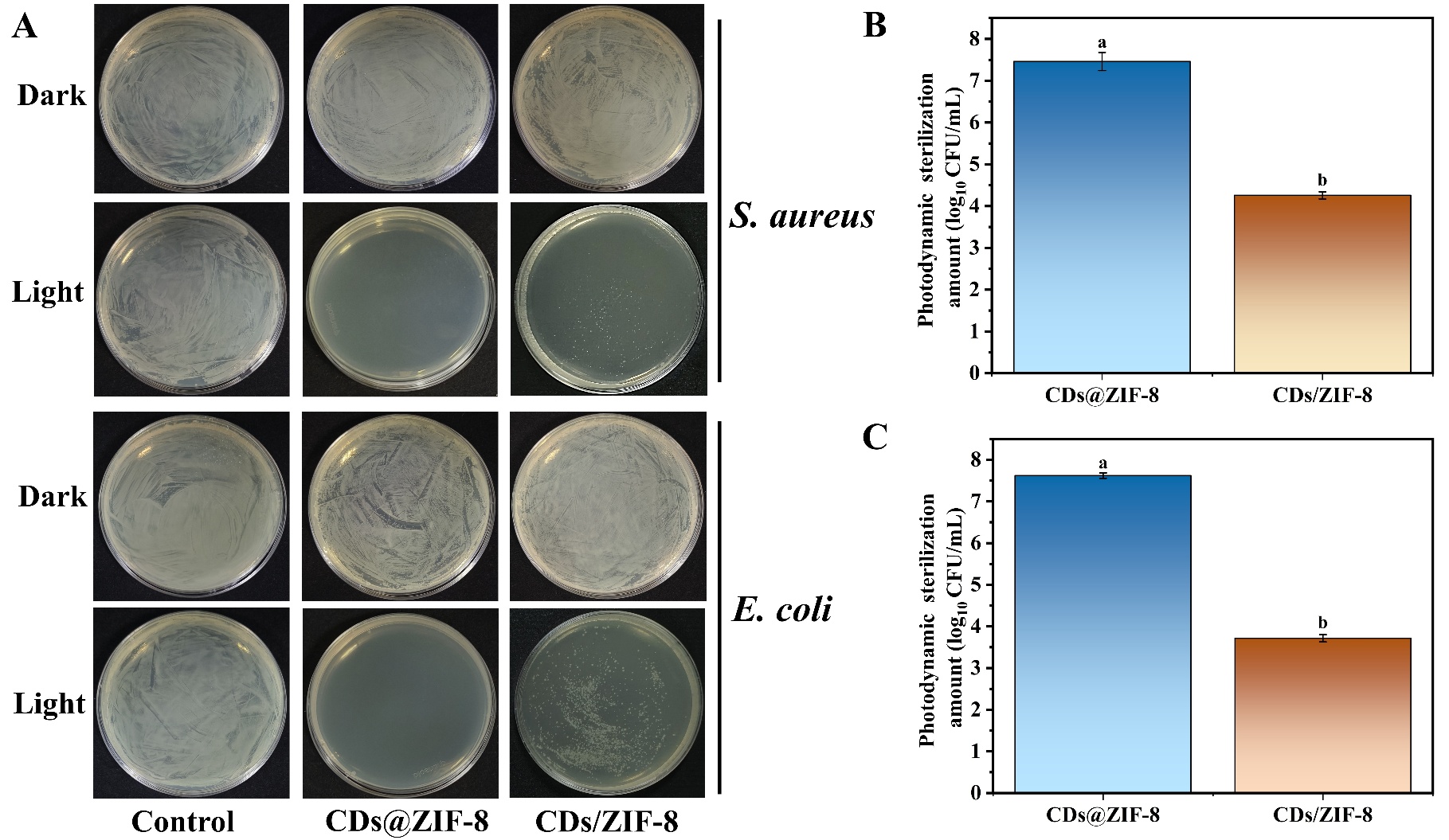


**Figure S10.** A) *S. aureus* and *E. coli* colony growth after treatment with CDs@ZIF-8 and CDs/ZIF-8 nanocomposites. Photodynamic sterilization amount of B) *S. aureus* and C) *E. coli* by CDs@ZIF-8 and CDs/ZIF-8 nanocomposites. Different letters (a-b) represent significant differences (**p* < 0.05).


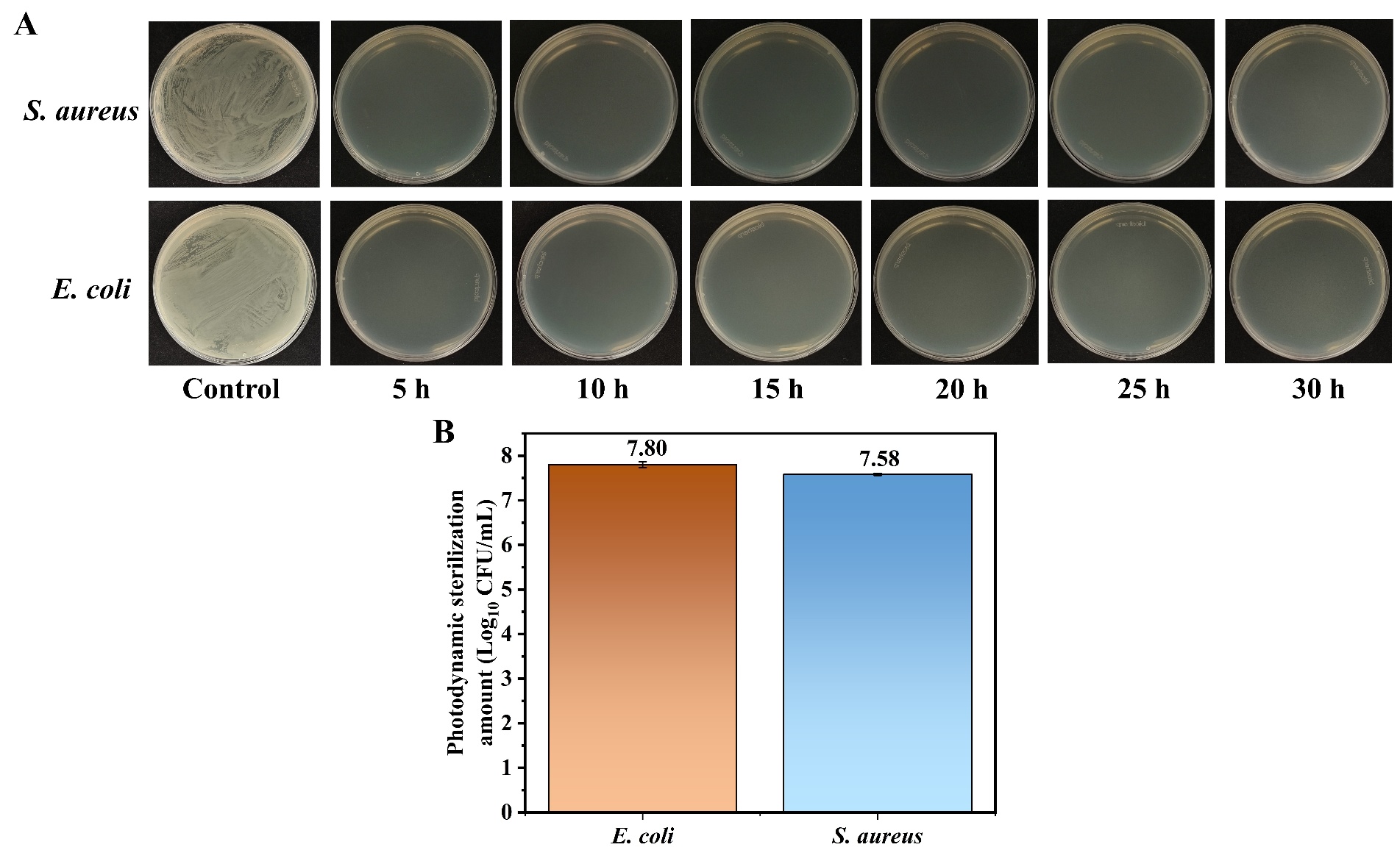


**Figure S11.** A) CDs@ZIF-8 nanocomposites were first exposed to continuous light for 5-30 h and then co-cultured with bacteria for photodynamic sterilization (3 h for *E. coli* and 2 h for *S. aureus*). B) The photodynamic sterilization amount of CDs@ZIF-8 nanocomposites to *E.coli* and *S.aureus*.


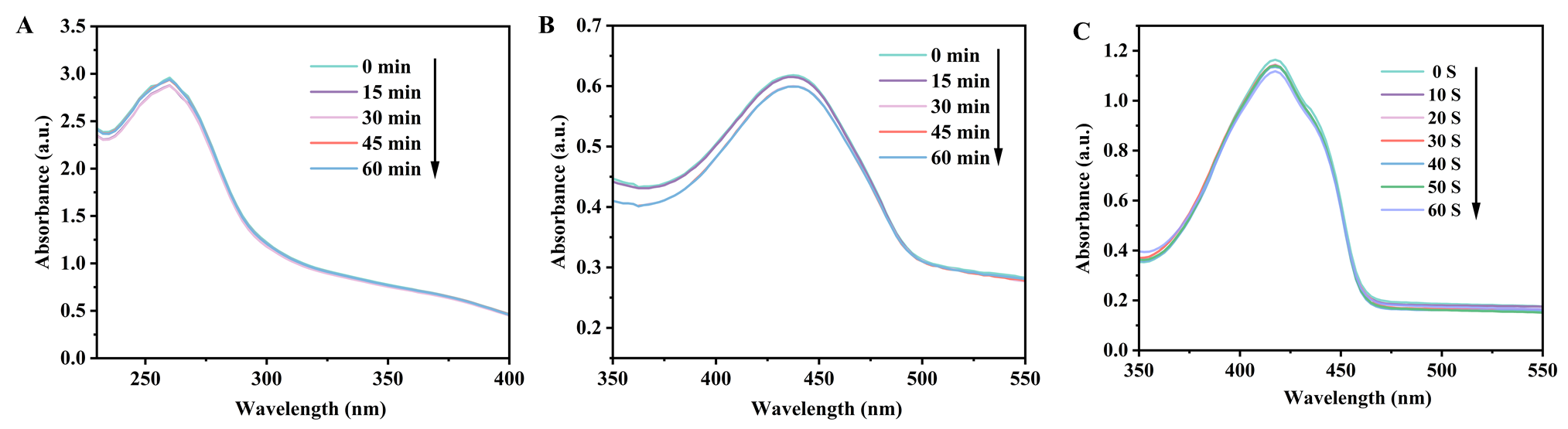


**Figure S12.** A) Determination of •O_2_^−^ by the NBT absorption under dark. B) Determination of ^1^O_2_ by the RNO absorption under dark. C) Determination of ^1^O_2_ by the DPBF fluorescent probe under dark.
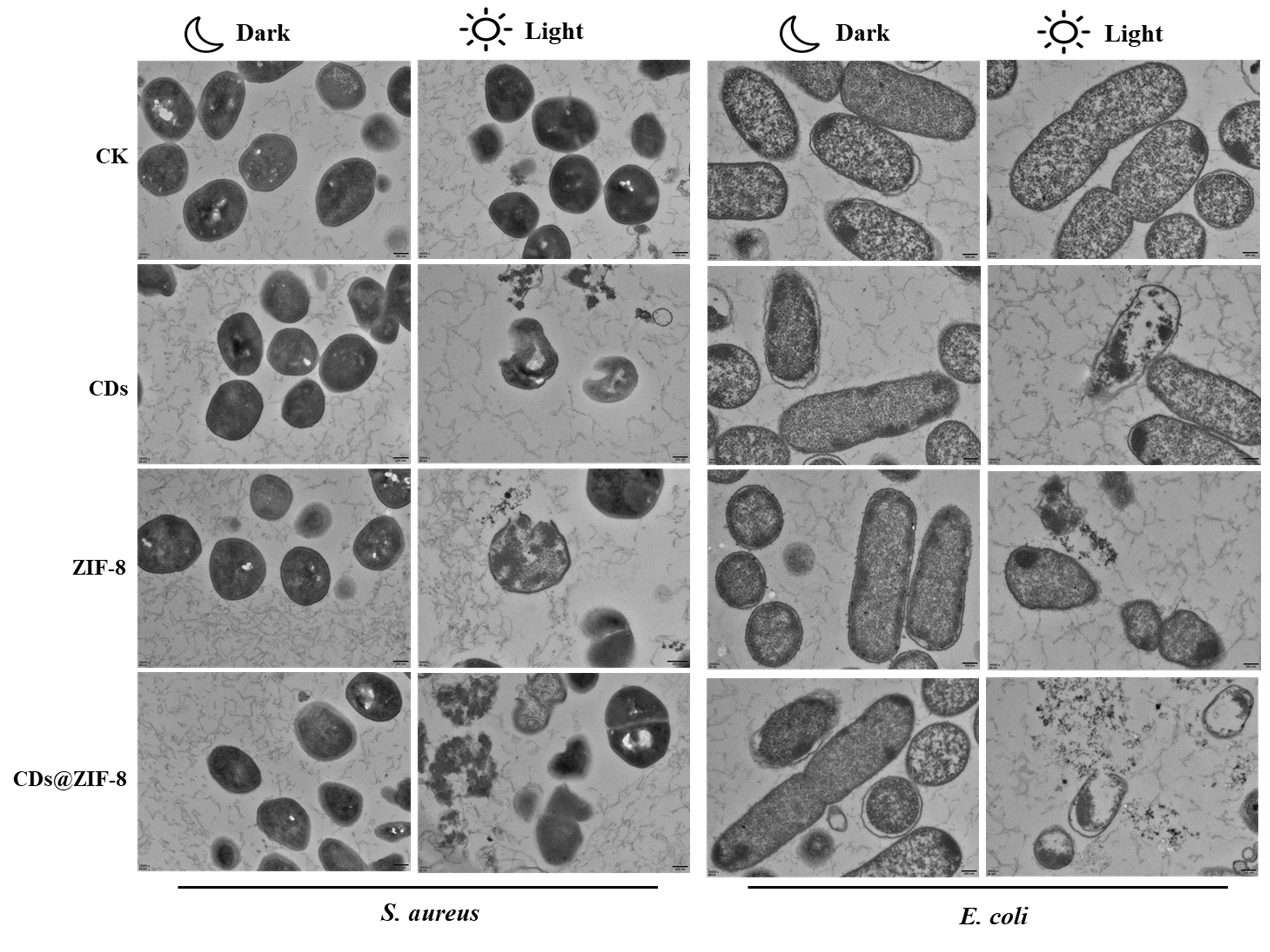


**Figure S13.** TEM micrographs of *S. aureus* and *E. coli* under dark and light, treated with normal saline, CDs, ZIF-8, and CDs@ZIF-8 nanocomposites, respectively (scar bar = 200 nm) (D), **p* < 0.05, ***p* < 0.01, ****p* < 0.001.


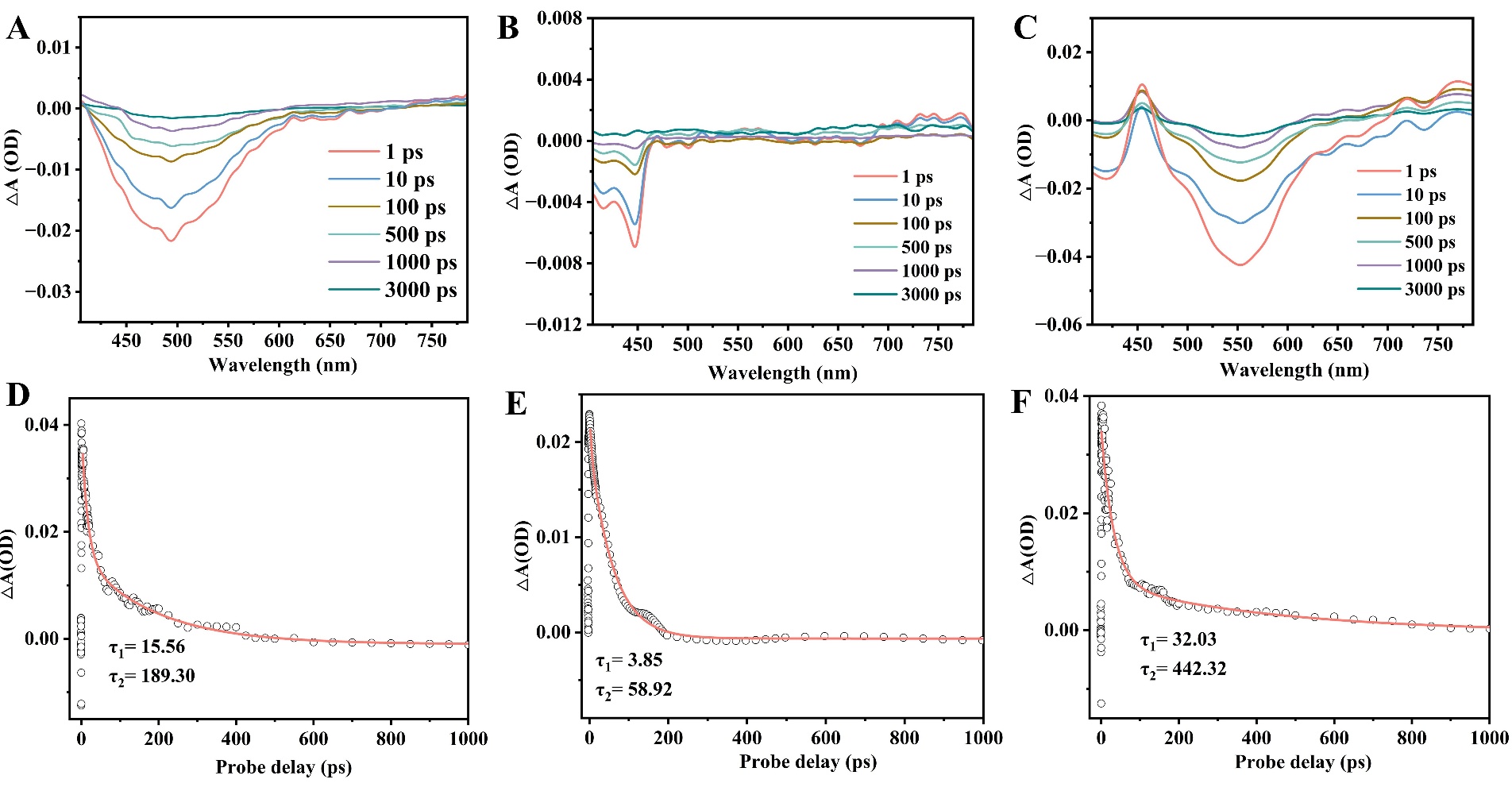


**Figure S14.** Ultrafast transient absorption (TA) spectra at different probe delays (pump at 345 nm) of A) CDs, B) ZIF-8 and C) CDs@ZIF-8. Representative TA kinetics at the probing wavelength at 450 nm of D) CDs, E) ZIF-8 and F) CDs@ZIF-8.


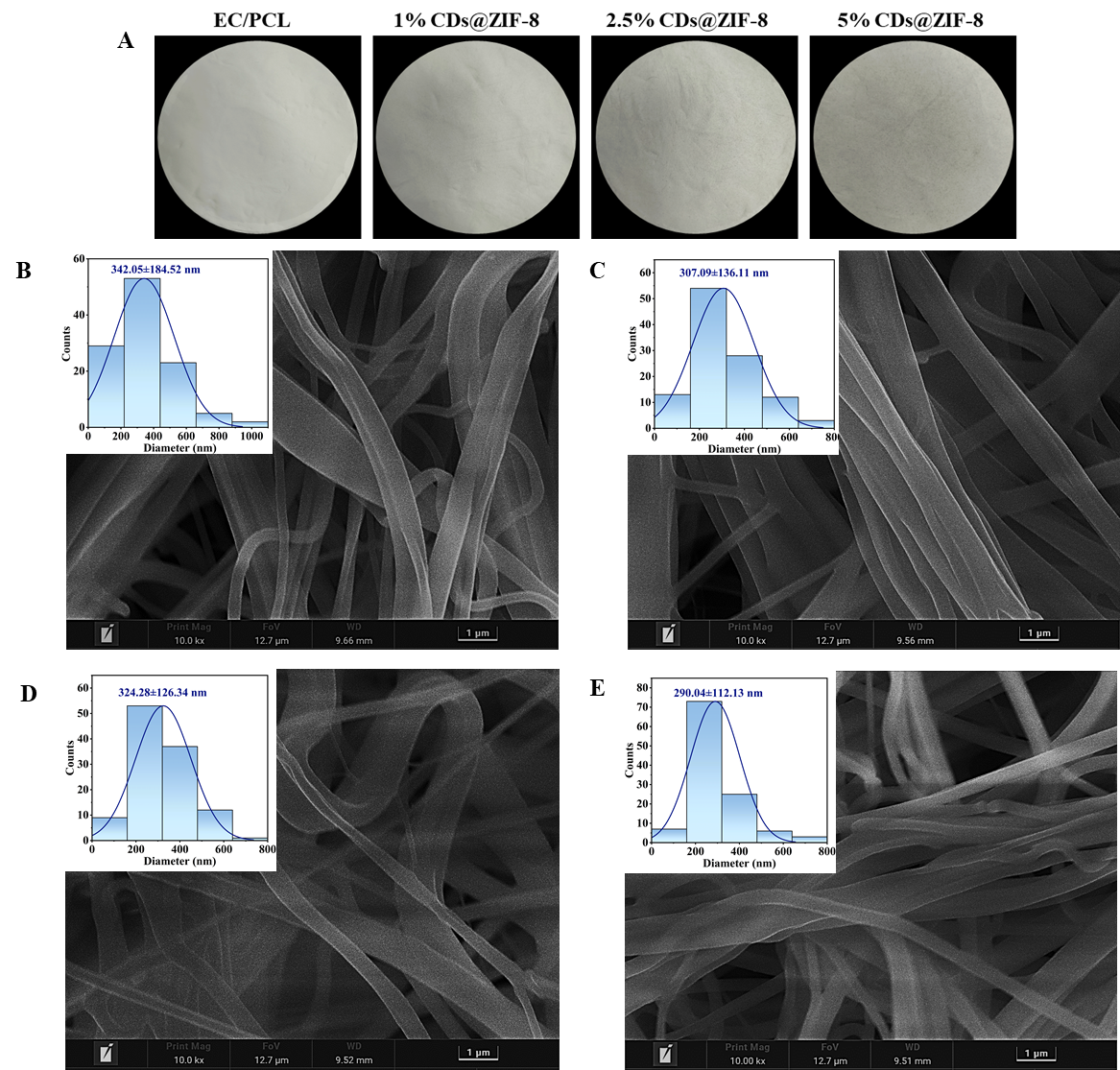


**Figure S15.** A) Digital photos of the PCL/EC nanofibrous films containing 0%, 1%, 2.5%, and 5% CDs@ZIF-8 nanocomposites, respectively. SEM images of PCL/EC nanofibrous films containing B) 0%, C) 1%, D) 2.5%, and E) 5% CDs@ZIF-8 nanocomposites, respectively.


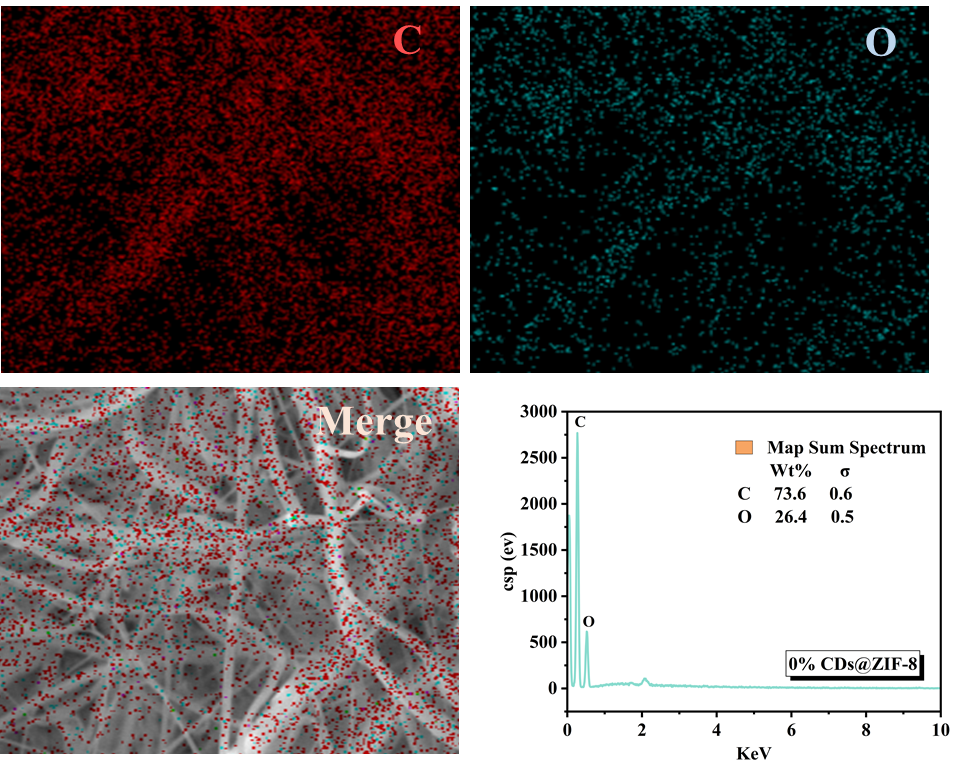


**Figure S16.** EDX spectroscopy elemental mapping images of PCL/EC nanofibrous films.


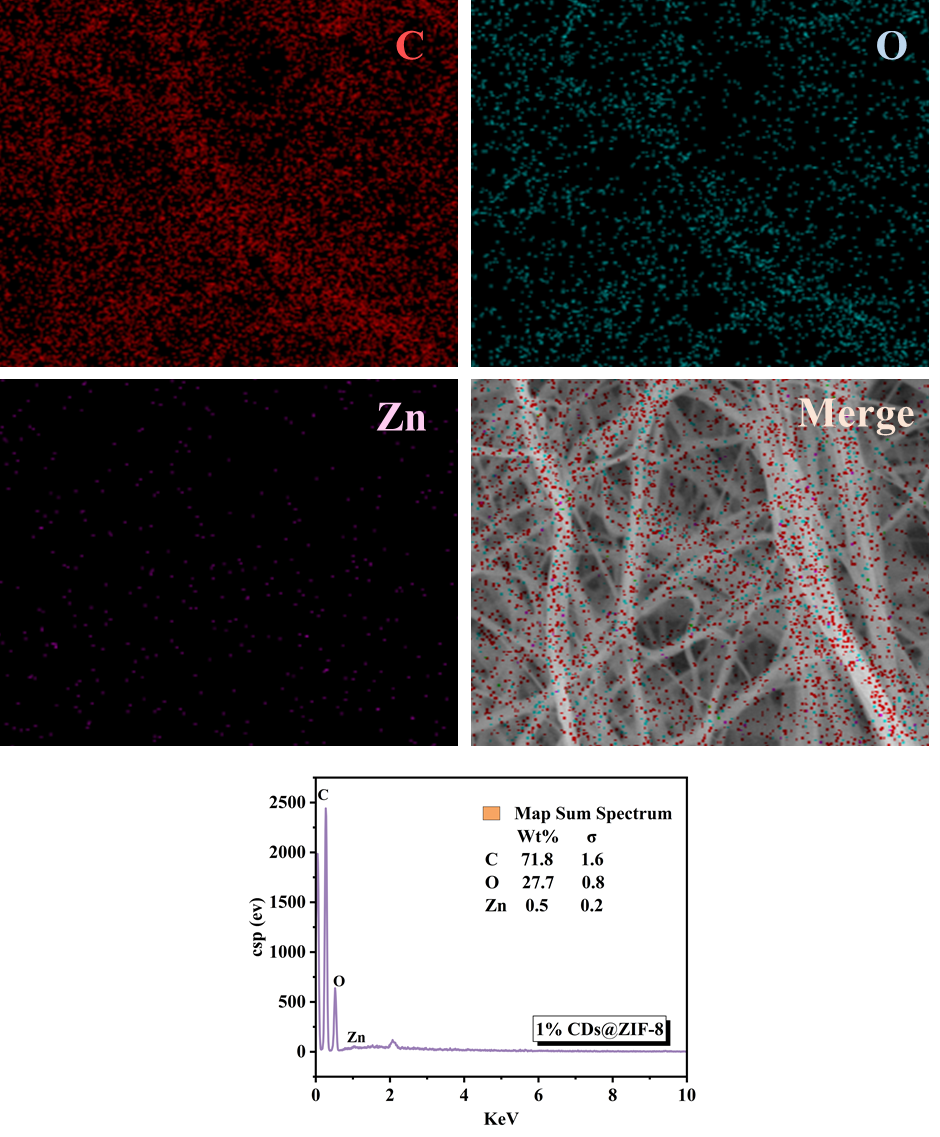


**Figure S17.** EDX spectroscopy elemental mapping images of 1% CDs@ZIF-8/PCL/EC nanofibrous films.


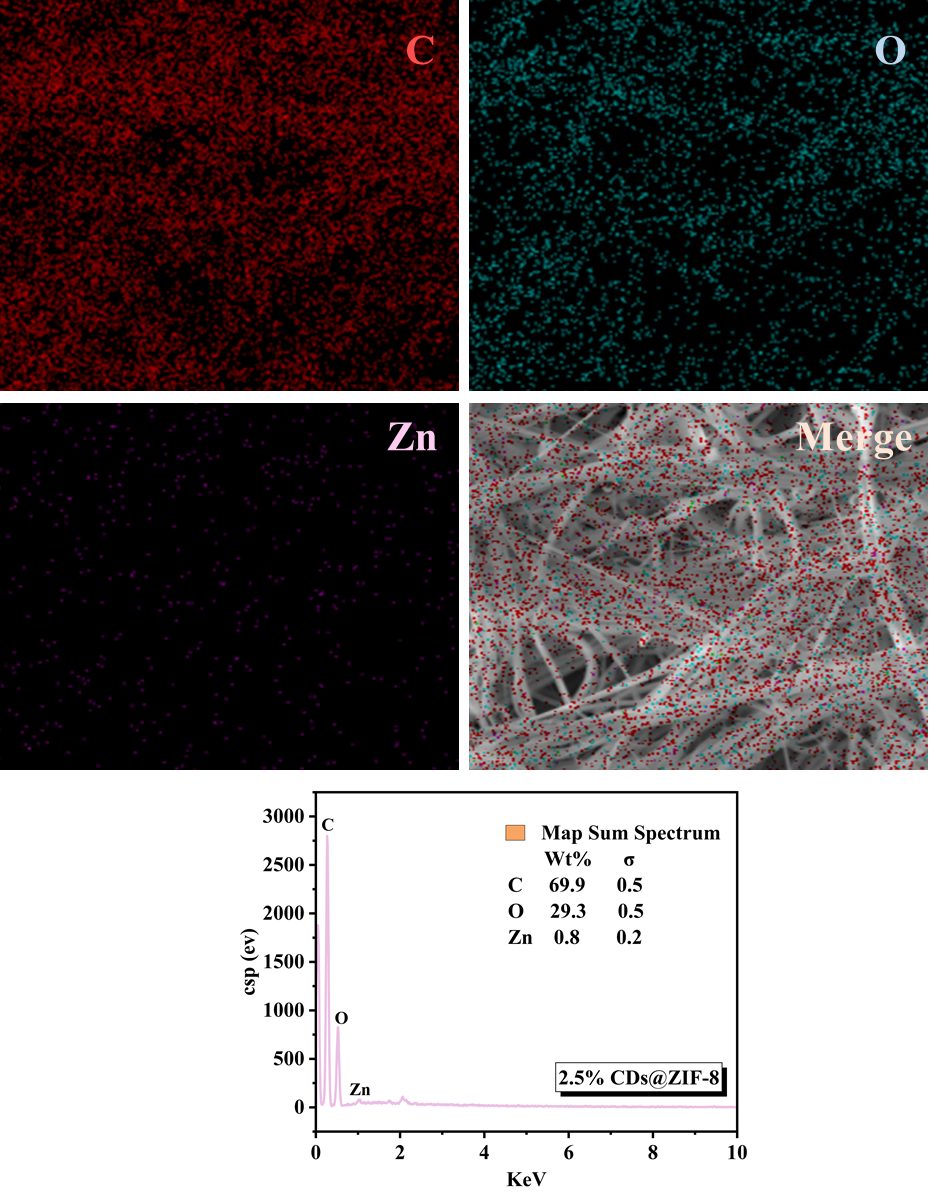


**Figure S18.** EDX spectroscopy elemental mapping images of 2.5% CDs@ZIF-8/PCL/EC nanofibrous films.


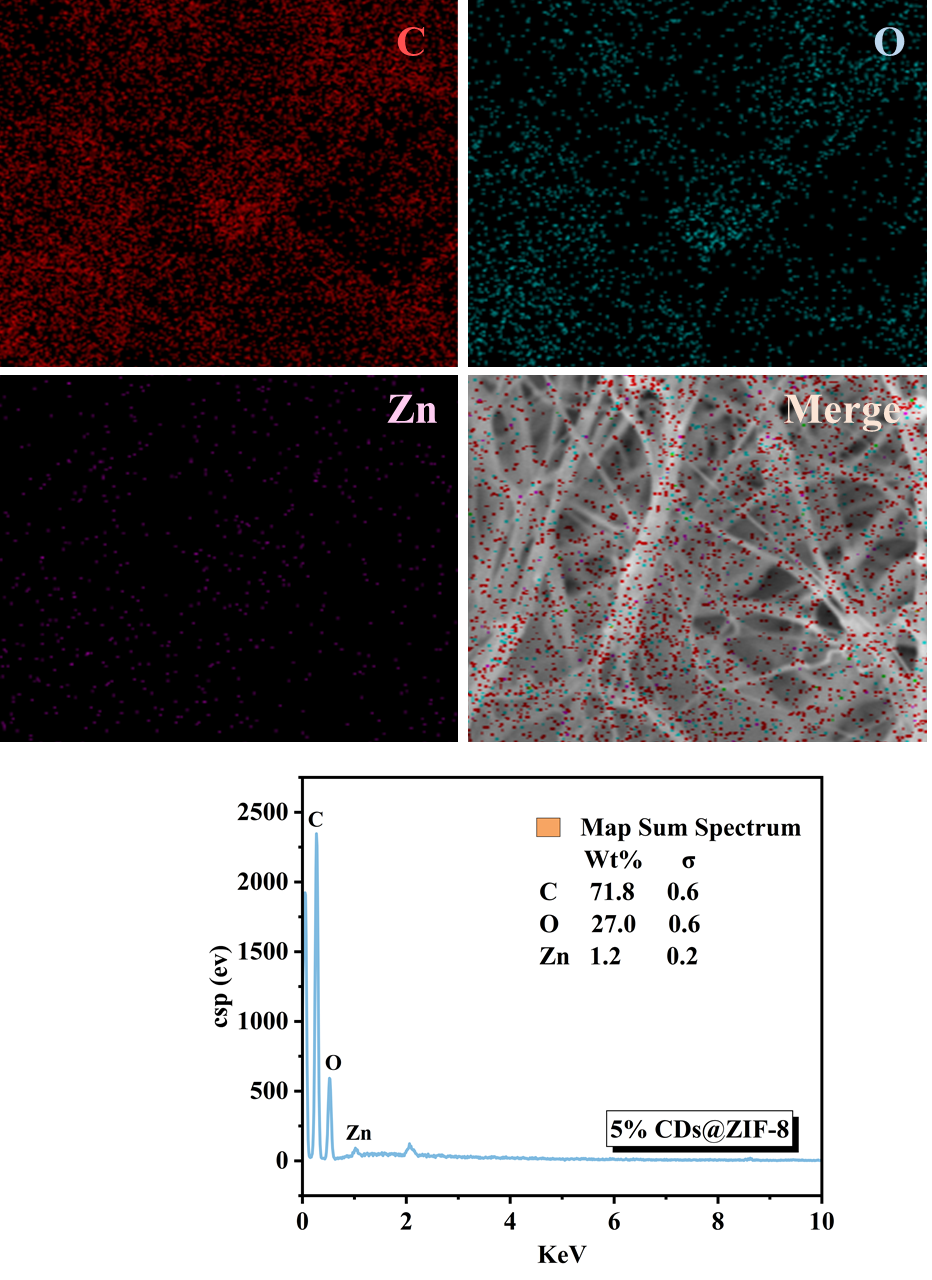


**Figure S19.** EDX spectroscopy elemental mapping images of 5% CDs@ZIF-8/PCL/EC nanofibrous films.

**
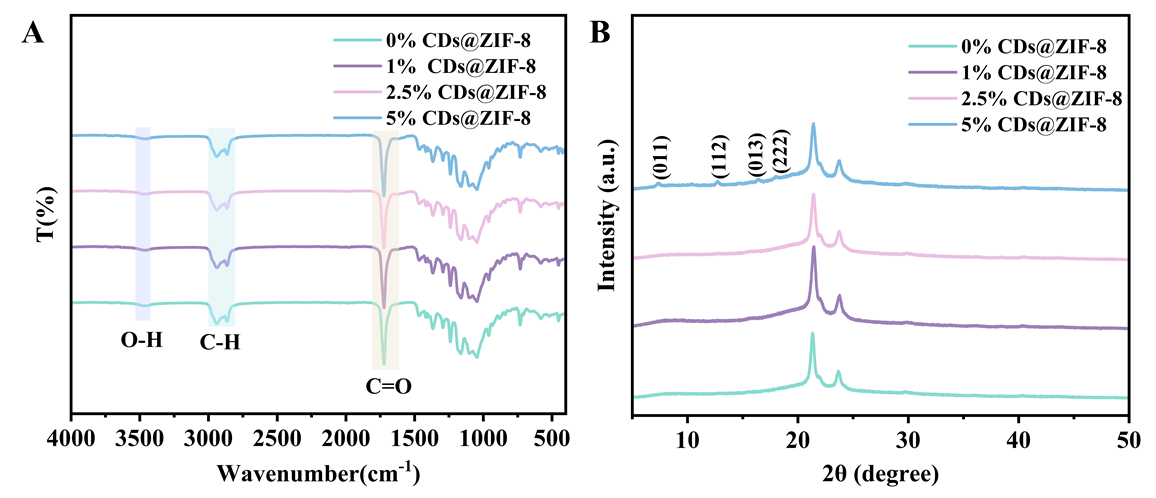
**

**Figure S20.** A) FT-IR spectra of the CDs@ZIF-8/PCL/EC nanofibrous films. B) XRD pattern of the PCL/EC/CDs@ZIF-8 nanocomposite films.


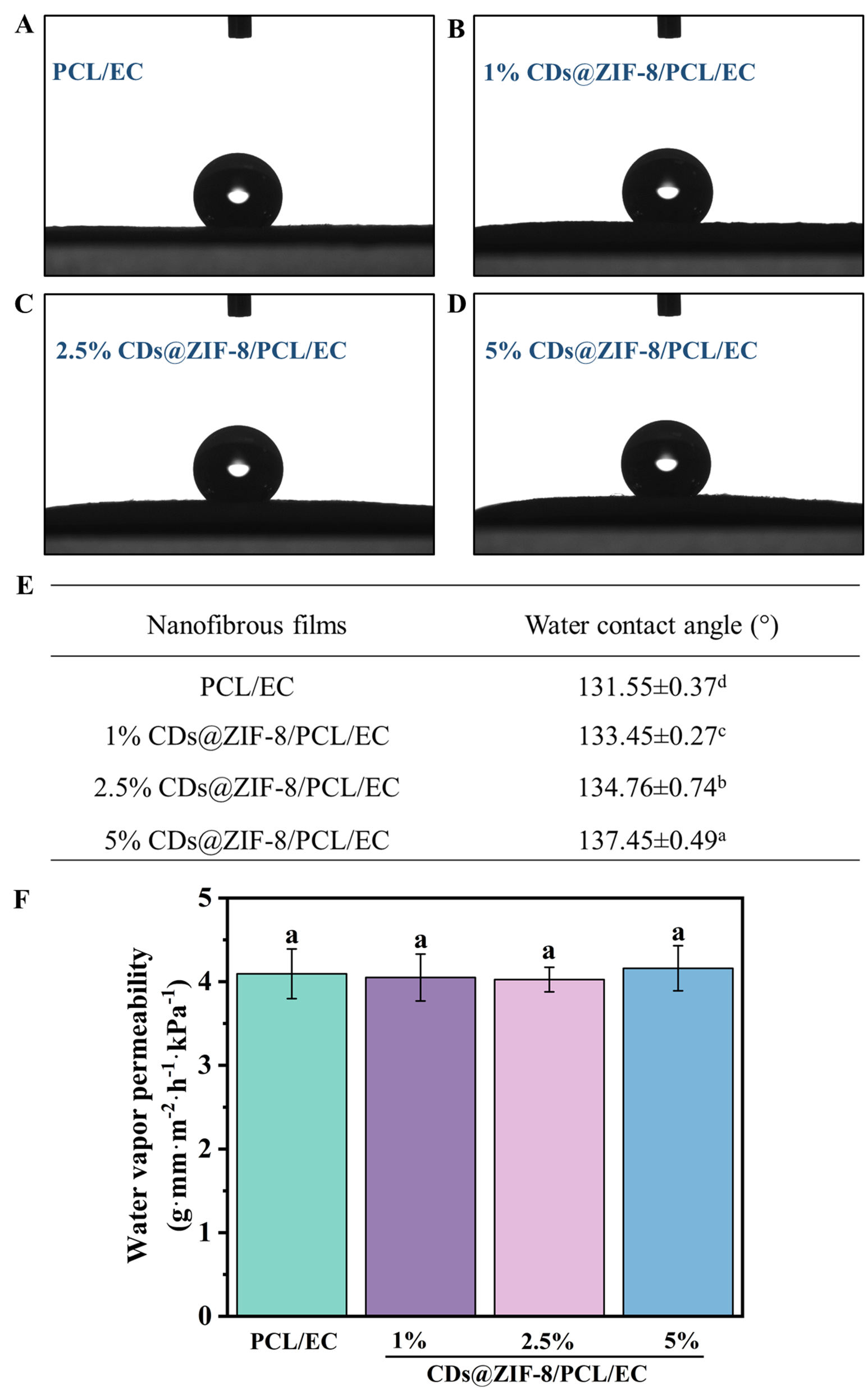


**Figure S21.** A) Contact angle image of the PCL/EC nanofibrous films, B) 1% CDs@ZIF-8/PCL/EC nanofibrous films, C) 2.5% CDs@ZIF-8/PCL/EC nanofibrous films, D) 5% CDs@ZIF-8/PCL/EC nanofibrous films, and E) the quantitative data of water contact angle. F) Water vapor permeability of nanofibrous films. Different letters (a-d) represent significant differences between groups (**p* < 0.05).


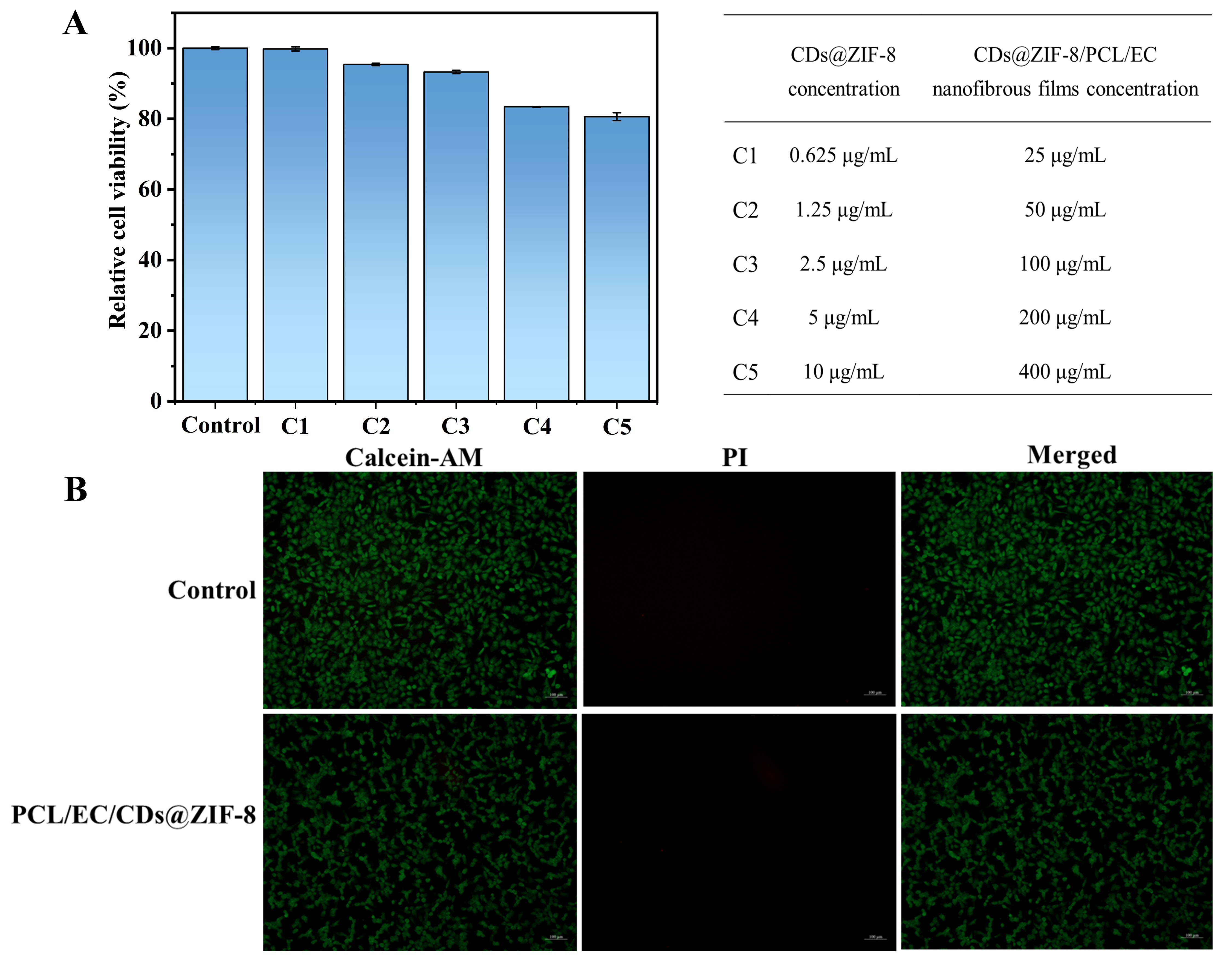


**Figure S22.** A) The relative viability of L929 cells co-incubated with CDs@ZIF-8/PCL/EC nanofibrous films at 24 h after 3 h of visible light irradiation and B) the fluorescence profiles of cells treated with 400 μg/mL CDs@ZIF-8/PCL/EC nanofibrous films (Live cells can be stained with Calcium-AM and show green fluorescence; dead cells can be stained with PI and show red fluorescence).


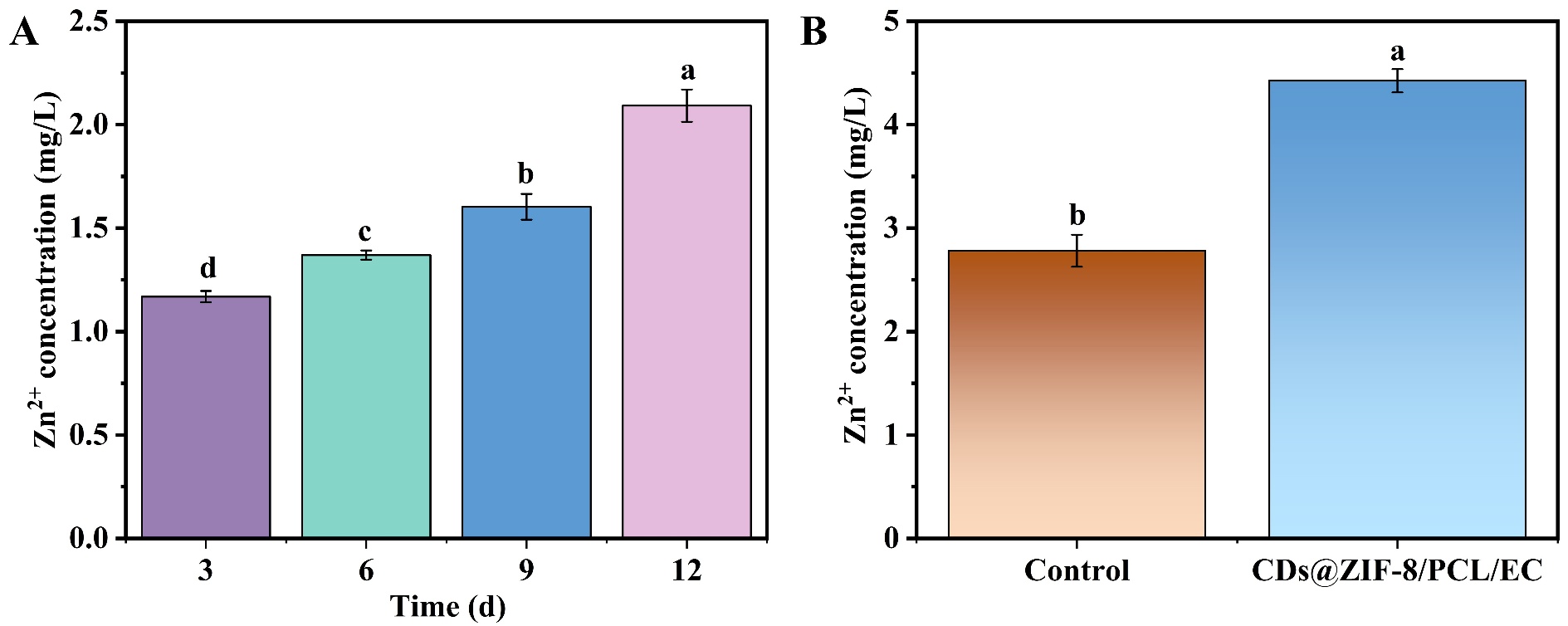


**Figure S23.** A) Zn^2+^ concentration in PBS solution. B) Zn^2+^ concentration in strawberries after 12 d of wrapping CDs@ZIF-8/PCL/EC nanofibrous films. Different letters (a-d) represent significant differences (*p* < 0.05).

**Table S1.** Sterilization capacity of different treatment groups at different illumination times.

|  | **Illumination**  **Time** | **Sterilization amount (Log_10_ CFU/mL)** | | | |
| --- | --- | --- | --- | --- | --- |
|  |  | **Blue light (CK)** | **CDs** | **ZIF-8** | **CDs@ZIF-8** |
| *E. coli* | 3h | 0.64 lg | 3.12 lg | 4.18 lg | 7.63 lg |
| *S. aureus* | 2h | 0.47 lg | 4.17 lg | 2.94 lg | 7.27 lg |

**Table S2.** DFT calculation of the electron transfer quantity.

|  | **Electron transfer quantity** |
| --- | --- |
| CDs | -0.0922 e |
| ZIF-8 | 0.0950 e |

**Table S3.** HOMO and LUMO values and HOMO-LUMO gaps from DFT calculations.

|  | **LUMO (eV)** | **HOMO (eV)** | **HOMO-LUMO gap (eV)** |
| --- | --- | --- | --- |
| CDs | -2.190 | -5.209 | 3.019 |
| ZIF-8 | -0.573 | -4.701 | 4.128 |
| CDs@ZIF-8 (Zn-O) | -2.791 | -5.011 | 2.220 |
| CDs@ZIF-8 (Zn-N) | -2.945 | -4.508 | 1.563 |

**Table S4.** DFT calculation of the O_2_ binding energies.

|  | **Binding energies of O_2_ ( kJ mol^−1^)** |
| --- | --- |
| CDs | -10.89 |
| ZIF-8 | -55.24 |
| CDs@ZIF-8 | -68.69 |

**Table** **S5.** TGA and DSC data of the CDs@ZIF-8/PCL/EC nanofibrous films.

|  | DSC parameters | | TGA parameters | | |
| --- | --- | --- | --- | --- | --- |
|  | **T_m_ (°C)** | **ΔH_m_ (J g^-1^)** | **T_5wt%_ (°C)^a^** | **T_max_ (%)^b^** | **W_red_ (%)^c^** |
| PCL/EC | 60.1 | 69.81 | 336.2 | 407.3 | 4.38 |
| 1% CDs@ZIF-8/PCL/EC | 58.9 | 58.84 | 327.9 | 394.6 | 4.30 |
| 2.5% CDs@ZIF-8/PCL/EC | 58.6 | 61.34 | 302.0 | 354.6 | 4.39 |
| 5% CDs@ZIF-8/PCL/EC | 59.4 | 62.87 | 311.6 | 337.2 | 5.58 |

^a^ T5_wt%_: temperature at 5% mass loss.

^b^ T_max_: temperature at the maximum weight loss rate.

^c^ W_red_: the residual weight at 600°C.

**Table S6.** Comparison of sterilizing properties and shelf-life extension of strawberry preservation materials.

| Preservation materials | *E. coli* sterilization | *S. aureus* sterilization | Extended shelf life | References |
| --- | --- | --- | --- | --- |
| CDs@ZIF-8/PCL/EC  nanofibrous films | 7.64 lg | 7.68 lg | 6 d | Our study |
| Tannic acid  /chitosan-citric acid preservation film | 1.17 lg | 0.97 lg | 5 d | [6] |
| Tormaldehyde cellulose  /polyvinyl alcohol/  Carboxymethyl cellulose composite films | / | / | 4 d | [7] |
| Chitosan coatings with turmeric and green tea extracts | / | / | 4 d | [8] |
| ZIF-67@tannic acid /chitin nanofiber films | 2.96 lg | 2.48 lg | / | [10] |
| Cu-tannic acid/  chitosan-gelatin nanocomposite films | / | / | 3 d | [11] |
| Zein-quercetin/  gelatin films | / | / | 2 d | [12] |
| Quaternary ammonium salt/TA modified chitosan/oxidized chitosan coating | 1.74 lg | 1.85 lg | 5 d | [13] |
| Carbon nanodots/silk  fibroin films | 4.00 lg | / | / | [14] |

**References**

[1] J. Guo, Y. Lu, A. Xie, G. Li, Z. Liang, C. Wang, X. Yang, S. Chen, Yellow-Emissive Carbon Dots with High Solid-State Photoluminescence, *Adv. Funct. Mater.* **2022**, *32*, 2110393.

[2] B. Tao, W. Zhao, C. Lin, Z. Yuan, Y. He, L. Lu, M. Chen, Y. Ding, Y. Yang, Z. Xia, K. Cai, Surface Modification of Titanium Implants By ZIF-8@Levo/LBL Coating for Inhibition of Bacterial-Associated Infection and Enhancement of In Vivo Osseointegration, *Chem. Eng. J.* **2020**, *390*, 124621.

[3] C. Shen, M. Wu, C. Sun, J. Li, D. Wu, C. Sun, Y. He, K. Chen, Chitosan/PCL Nanofibrous Films Developed by SBS to Encapsulate Thymol/HPΒCD Inclusion Complexes for Fruit Packaging, *Carbohydr. Polym.* **2022**, *286*, 119267.

[4] C. Shen, Y. Ma, D. Wu, P. Liu, Y. He, K. Chen, Preparation of Covalent Organic Framework-Based Nanofibrous Films with Temperature-Responsive Release of Thymol for Active Food Packaging, *Food Chem.* **2023**, *410*, 135460.

[5] M. Wu, Z.-A. Deng, C. Shen, Z. Yang, Z. Cai, D. Wu, K. Chen, Fabrication of Antimicrobial PCL/EC Nanofibrous Films Containing Natamycin and Trans-Cinnamic Acid by Microfluidic Blow Spinning for Fruit Preservation, *Food Chem.* **2024**, *442*, 138436.

[6] L. Chang, L. Xu, Z. Yang, L. Liu, D. Qiu, Antibacterial and Antioxidative Biogenic Films for Room-Temperature Strawberry Preservation, *Food Chem.* **2023**, *405*, 134893.

[7] X. Fu, S. Xu, M. Ma, Preparation of DCNC Chemically Cross-Linked CMC/PVA Composite Film for Sustainable and Strawberry Preservation Active Packaging, *Int. J. Biol. Macromol.* **2025**, *306*, 141671.

[8] C. Yang, J.-H. Lu, M.-T. Xu, X.-C. Shi, Z.-W. Song, T.-M. Chen, D. D. Herrera-Balandrano, Y.-J. Zhang, P. Laborda, M. Shahriar, S.-Y. Wang, Evaluation of Chitosan Coatings Enriched with Turmeric and Green Tea Extracts on Postharvest Preservation of Strawberries, *LWT* **2022**, *163*, 113551.

[9] C. Yin, X. Ding, Z. Lin, J. Cao, W. Shi, J. Wang, D. Xu, D. Xu, Y. Liu, G. Liu, Preparation and Characterization of Quercetin@ZIF-L/GO@Agnps Nanocomposite Film for Room-Temperature Strawberry Preservation, *Food Chem.* **2024**, *450*, 139411.

[10] M. J. Khan, F. Hafeez, M. R. Islam, C. Zhu, Y. Xianyu, Advanced Antibacterial Packaging for Food Preservation Through Multifunctional Metal–Organic Framework Nanocomposite, *Small*, **2025**, 2501111.

[11] W. Sheng, L. Yang, Y. Yang, C. Wang, G. Jiang, Y. Tian, Photo-Responsive Cu-Tannic Acid Nanoparticle-Mediated Antibacterial Film for Efficient Preservation of Strawberries, *Food Chem.* **2025**, *464*, 141711.

[12] X. Xu, D. Dai, H. Yan, J. Du, Y. Zhang, T. Chen, Enhancing Mechanical and Blocking Properties of Gelatin Films Using Zein-Quercetin Nanoparticle and Applications for Strawberry Preservation, *Food Chem.* **2025**, *464*, 141895.

[13] C. He, L. Yuan, S. Bi, C. Zhou, Q. Yang, J. Gu, B. Yan, J. He, Modified Chitosan-Based Coating/Packaging Composites with Enhanced Antibacterial, Antioxidant, and UV-Resistant Properties for Fresh Food Preservation, *ACS Appl. Mater. Interfaces* **2024**, *16*, 48352.

[14] W.-B. Zhao, Y. Wang, F.-K. Li, R. Guo, F.-H. Jiao, S.-Y. Song, S.-L. Chang, L. Dong, K.-K. Liu, C.-X. Shan, Highly Antibacterial and Antioxidative Carbon Nanodots/Silk Fibroin Films for Fruit Preservation, *Nano Lett.* **2023,** *23*, 11755.
